# Folliculin depletion results in liver cell damage and cholangiocarcinoma through MiT/TFE activation

**DOI:** 10.1038/s41418-025-01486-8

**Published:** 2025-04-06

**Authors:** Bruno Maria Custode, Francesco Annunziata, Felipe Dos Santos Matos, Valentina Schiano, Veronica Maffia, Milena Lillo, Rita Colonna, Rossella De Cegli, Andrea Ballabio, Nunzia Pastore

**Affiliations:** 1 Telethon Institute of Genetics and Medicine (TIGEM), Pozzuoli (NA), Italy; 2 Jan and Dan Duncan Neurological Research Institute, Texas Children Hospital, Houston, TX USA; 3 Department of Molecular and Human Genetics, Baylor College of Medicine, Houston, TX USA; 4 Department of Translational Medicine, Medical Genetics, Federico II University, Naples, Italy

**Keywords:** Cancer, Gene expression

## Abstract

Mutations in the tumor suppressor gene Folliculin (*FLCN*) are responsible for Birt-Hogg-Dube’ (BHD) syndrome, a rare inherited condition that predisposes affected individuals to skin tumors, pulmonary cysts, and kidney tumors. FLCN regulates key cellular pathways, including TFEB, TFE3, and mTORC1, which are critical for maintaining cell homeostasis. Loss of FLCN leads to both hyperactivation of mTORC1 and constitutive activation of TFEB and TFE3, contributing to tumorigenesis. While previous studies showed that Flcn liver-specific conditional knockout (Flcn^LiKO^) mice are protected from developing liver fibrosis and damage upon high-fat diet exposure, the potential role of FLCN loss in liver carcinogenesis remained unexplored. Here, we demonstrate that hepatic loss of FLCN in mice results in cancer associated with inflammation and fibrosis with features of cholangiocarcinoma (CCA). This phenotype emerges in mice over 90-week-old, with a male predominance. Moreover, Flcn^LiKO^ mice are more prone to develop diethylnitrosamine (DEN)- or 3,5-diethoxycarbonyl-1,4-dihydrocollidine (DDC)- induced liver tumors with heterogenous histological features. Notably, depletion of TFE3, but not TFEB, in the liver of Flcn^LiKO^ mice fully rescues the cancer phenotype and normalized mTORC1 signaling, highlighting TFE3 as the primary driver of liver cancer and mTORC1 hyperactivity in the absence of FLCN.

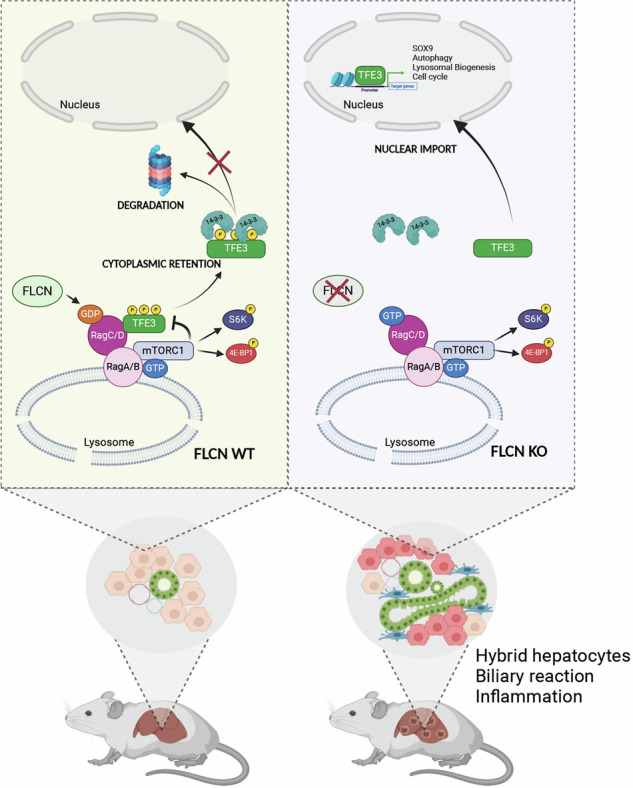

## Introduction

Primary liver cancer is the third leading cause of cancer-related death worldwide [1]. Hepatocellular carcinoma (HCC) and cholangiocellular carcinoma (CCA) are the most frequent and aggressive primary liver tumors, with liver transplantation being the only curative option [2, 3]. The risk of developing these malignancies is heavily influenced by environmental factors, such as viral infections, alcohol consumption, and diet [4]. Both HCC and CCA develop through a multi-step process, often initiated by liver damage leading to chronic inflammation and cycles of necrosis and regeneration [5]. Although several studies have described the molecular mechanisms involved in HCC and CCA development [6, 7], several knowledge gaps still need to be addressed. Moreover, patients with advanced HCC and CCA have limited treatment options, and chemotherapy provides minimal survival benefit. Therefore, identifying novel therapeutic targets for these devastating diseases is critical.

The MiT/TFE transcription factors (TFs) TFEB and TFE3 are recognized as master regulators of lysosomal biogenesis and autophagy [8, 9]. MiT/TFE factors were originally described as oncogenes as they are involved in chromosomal translocations and gene amplifications associated with various types of cancers such as melanoma, renal cell carcinoma, and alveolar sarcoma [10]. Furthermore, we recently reported that liver-specific TFEB overexpression in mice causes liver damage, impaired cell differentiation, and CCA development in a time- and dose-dependent manner, largely through the regulation of SOX9 expression [11]. We also described how autophagy activation, due to TFEB overexpression, plays a critical role in cilia disassembly in CCA, favoring tumor growth [12].

The activity of MiT/TFE TFs is negatively regulated by Folliculin (FLCN), which is a GTPase Activating Protein (GAP) for RagC and D GTPases. Active RagC and RagD enable substrate-specific phosphorylation and cytoplasmic retention of TFEB and TFE3 by mTORC1 [13]. Conversely, loss of FLCN leads to dephosphorylation and nuclear translocation of TFEB and TFE3, which in turn drive mTORC1 hyperactivation through a previously described MiT-TFE/mTORC1 feedback loop [14].

Loss-of-function mutations in the FLCN gene cause Birt-Hogg-Dube’ (BHD) syndrome, an autosomal dominant disorder characterized by the presence of multiple benign cutaneous tumors known as fibrofolliculomas, pulmonary cysts often leading to pneumothorax, as well as kidney cysts and tumors [15, 16]. The cystic and tumorigenic phenotypes observed in kidney-specific Flcn^KO^ mice are completely rescued by TFEB deletion [13]. In addition, silencing of either TFEB or TFE3 rescued tumorigenesis in mouse xenografts generated using a cell line derived from a kidney cancer patient with BHD syndrome [17], indicating that TFEB and TFE3 are the main drivers of tumorigenesis in multiple tissues.

Interestingly, FLCN loss has been described as beneficial in various tissues, including skeletal muscle [18], adipose tissue [19, 20], and liver [21, 22], likely due to the constitutive activation of TFEB and TFE3. Recent studies demonstrated that liver-specific Flcn conditional knockout (KO) mice are protected from the consequences of a high-fat diet, including Non-Alcoholic Fatty Liver Disease (NAFLD), fibrosis, and inflammation [21, 22]. In this study, we show that liver-specific Flcn conditional KO mice exhibit impaired cell homeostasis upon liver injury and recovery, along with a late onset, gender-dependent hepatocarcinogenic phenotype primarily driven by TFE3 activation.

## Results

### Liver-specific depletion of FLCN leads to liver injury in young mice

**Fig. 1:**
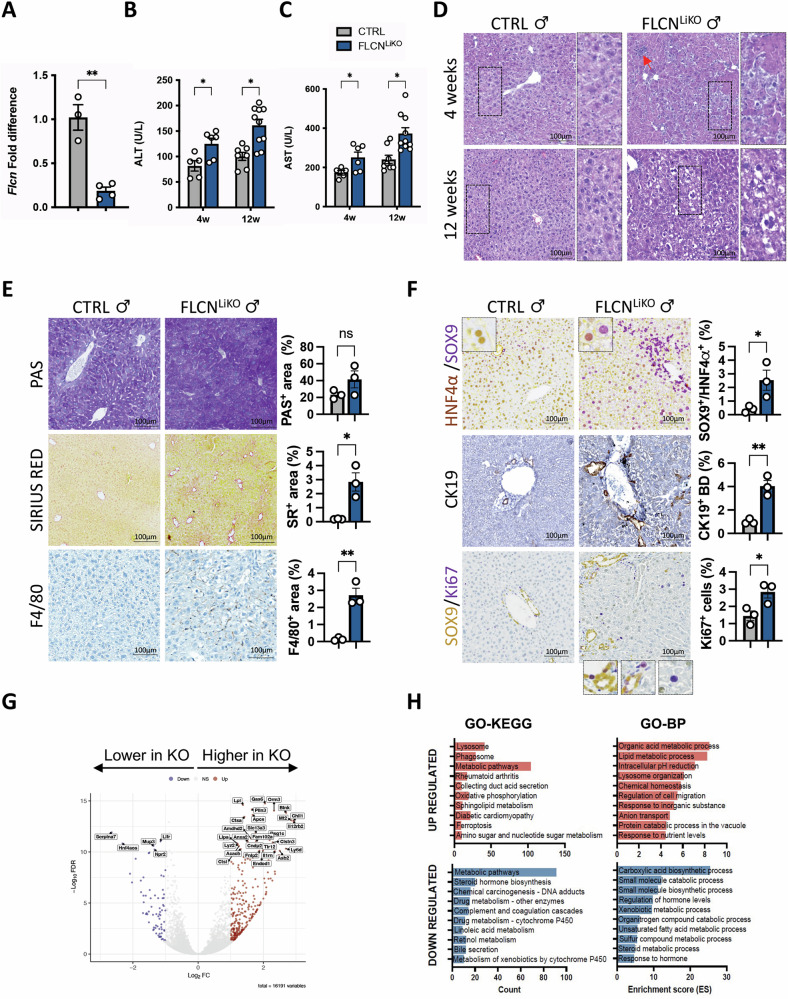
Characterization of liver-specific Flcn knockout male mice. **A** *Flcn* expression levels after CRE recombination in 12-week-old Flcn^LiKO^ and control mice (n = 3 CTRL and n = 4 Flcn^LiKO^). Serum ALT (**B**) and AST (**C**) levels in Flcn^LiKO^ (n = 6 at 4 weeks and n = 10 at 12 weeks) and control mice (n = 5 at 4 weeks and n = 7 at 12 weeks) at the indicated time points. **D** H&E staining of liver section from mice of the indicated genotypes at 4 and 12 weeks of age, showing clear cells and hepatocyte ballooning. Red arrows indicate immune infiltrates. **E**, **F** PAS and Sirius red staining (**E**), along with immunostaining for the indicated markers (**F**) in liver sections from 12-week-old Flcn^LiKO^ and control male mice, with relative quantification (n = 3 per group). **G** Volcano plot of differentially expressed genes (DEGs) between 12-week-old male Flcn^LiKO^ and control mice. **H** Gene Ontology (GO) analysis, including KEGG and Biological Process terms, highlighting significantly upregulated (red) and downregulated (blue) pathways in Flcn^LiKO^ mice compared to control (FDR < 0.05). All the data refer to male mice. Each dot represents an individual mouse. Data are mean ± standard error. Statistical analysis: Student *t*-test *p-value < 0.05, **p-value < 0.01.

To investigate the role of FLCN in liver homeostasis, we generated a liver-specific Flcn KO mouse line (Flcn^LiKO^) by crossing Flcn^fl/fl^ and Alb-Cre mouse lines. To explore the effect of Flcn depletion, we collected livers from Flcn^LiKO^ and control male mice at 4- and 12 weeks of age for analysis. As previously reported [11], Alb-Cre results in Cre recombination in 99% of liver epithelial cells leading to a strong reduction of Flcn expression at the mRNA level in adult mice (Fig. 1A). Although no differences were observed in body weight (BW) (Fig. S1A) or in liver-to-body weight (LW/BW) ratio at the indicated time points (Fig. S1B), Flcn^LiKO^ male mice exhibited signs of liver damage as early as 4 weeks of age. This was evident from elevated serum alanine aminotransferases (ALT) and aspartate aminotransferases (AST) levels (Fig. 1B, C) and histological features including dysplastic and ballooned hepatocytes (large cells with clear cytoplasm) and immune cell infiltrations (Fig. 1D). Period Acid-Schiff (PAS) staining, Sirius red staining, and immunostaining for the specific macrophage marker F4/80 indicated abnormal glycogen accumulation, mild fibrotic changes, and inflammation, respectively (Fig. 1E).

Given our previous findings on TFEB overexpression and its impact on liver cell fate and regeneration [11], we next examined whether FLCN depletion results in similar effects in the liver due to constitutive activation of TFEB and TFE3 [23, 24]. We observed immunostaining for SOX9, a progenitor and cholangiocyte marker, in hepatocytes, suggesting that Flcn loss induced ectopic expression of SOX9 and the appearance of hybrid hepatocytes exhibiting features of both hepatocytes and progenitor cells in young mice (Figs. 1F and S1C). Furthermore, CK19 immunostaining, a cholangiocyte marker, revealed enlarged bile ducts with a strong ductular reaction in Flcn^LiKO^ mice (Fig. 1F). Ki67 immunostaining showed cell proliferation in both hepatocytes and SOX9^+^ cells in the portal areas in Flcn^LiKO^ mice compared to controls (Fig. 1F).

Interestingly, Flcn^LiKO^ female mice exhibited a milder phenotype compared to males with a slight but not significant increase in serum ALT and AST levels at 4- and 12- weeks of age (Fig. S2A) and vacuolated hepatocytes (Fig. S2B), and a mild but not significant increase in glycogen and collagen deposition, as demonstrated by PAS and Sirius red staining (Fig. S2C). Notably, immunostaining for SOX9 and CK19 showed expansion of progenitor cells and a biliary reaction in females, with SOX9^+^ signal confined to the portal tract, and no evidence of SOX9^+^ hepatocytes (Fig. S2D).

RNA-seq analysis performed from liver samples of male and female Flcn^LiKO^ and control mice at 12 weeks of age (GSE269752) revealed 1,126 differentially expressed genes (DEGs), of which 646 were induced and 480 were inhibited in Flcn^LiKO^ male mice (Figs. 1G and S3A). Gene Ontology enrichment analysis (GOEA) and KEGG pathway analysis showed that upregulated transcripts were mostly enriched in lysosome, phagosome, and oxidative phosphorylation categories [9, 25, 26], while inhibited transcripts were enriched in pathways related to metabolism, steroid hormone biosynthesis, and drug metabolism, among others (Fig. 1H and Table S1). In contrast, the female dataset at 12 weeks of age showed a weaker transcriptional response, with 327 DEGs, with 136 induced and 191 inhibited (Figs. S2E and S3B). Functional analysis of these showed that the induced transcripts were mainly involved in the stress response to external stimulus and nutrient levels, defense response, and cell death, while the inhibited transcripts were in metabolic pathways including organic acid metabolic process, carboxylic acid biosynthetic process, and regulation of lipid metabolism (Fig. S2F and Table S2). Interestingly, Heat map for hepatocyte- and progenitor/cholangiocyte-specific markers showed a reduction in the expression levels of hepatocyte-specific genes with a concomitant upregulation of progenitor-specific markers more pronounced in male than in female Flcn^LiKO^ mice, confirming the abnormal differentiation of hepatocytes (Fig. S3C, D).

Comparison of DEGs between male and female Flcn^LiKO^ mice revealed 84 commonly upregulated and 66 commonly downregulated genes (Fig. S3E and Table S3). Both groups showed enrichment in lysosome and PPAR signaling pathways, while metabolic and drug metabolism pathways were inhibited, correlating with impaired hepatocyte function (Fig. S3F and Table S4).

The observed differences in gene expression in males and females was coherent with the observed liver phenotype.

### Sexual dimorphism is due to gender-specific differential activation of TFE3 and TFEB

**Fig. 2:**
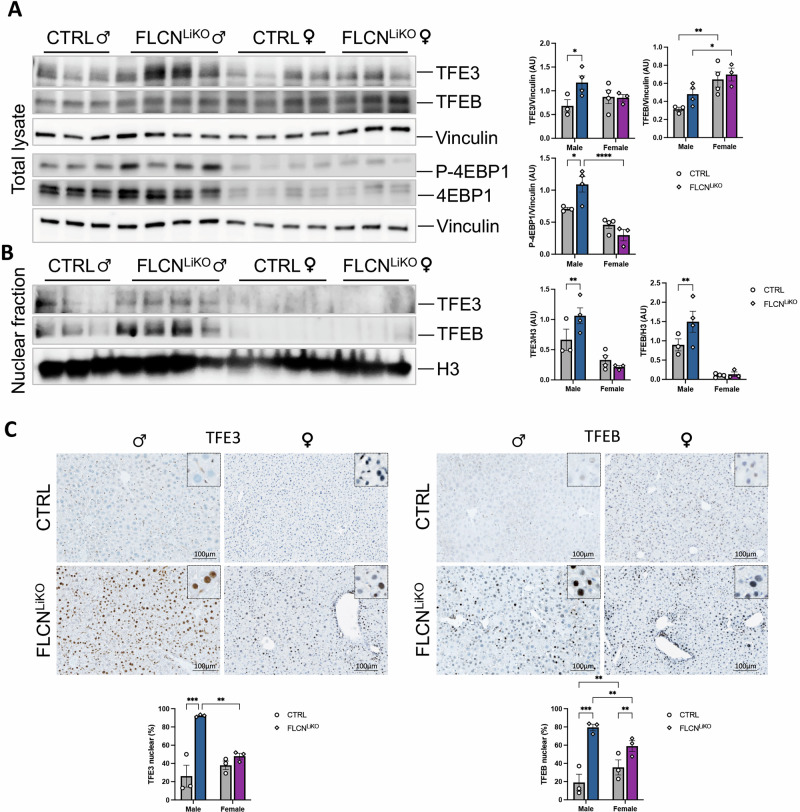
Sexual dimorphism in TFE3 and TFEB activation in Flcn-depleted livers. **A** Immunoblot analysis of TFE3, TFEB, and mTORC1 target P-4EBP1 in total liver lysate from male and female Flcn^LiKO^ and control mice at 12 weeks of age, with relative quantifications (n = 3-4 per group). Nuclear fraction analysis with quantifications (n = 3-4 per group) (**B**) and immunostaining with relative quantifications (n = 3 per group) (**C**), showing TFE3 and TFEB nuclear localization in male and female Flcn^LiKO^ and control mice. Each dot represents an individual mouse. Blue bars indicate male, and purple bars indicate female mice. Data are mean ± standard error. Statistical analysis: One-way ANOVA: *p-value < 0.05; **p-value < 0.01; ***p-value < 0.001 ; ****p-value < 0.0001.

We next investigated the mechanism underlying the sexual dimorphism observed in male and female Flcn^LiKO^ mice. Immunoblot analysis of total liver lysate revealed significantly higher levels of TFE3 protein in male Flcn^LiKO^ mice compared to females (Fig. 2A). In contrast, TFEB protein levels were higher in female Flcn^LiKO^ and control mice than in males (Fig. 2A). Nevertheless, analysis of nuclear fractions (Fig. 2B) and immunostaining of liver sections from Flcn^LiKO^ and control mice (Fig. 2C) showed that both TFE3 and TFEB were predominantly localized to the nucleus in male Flcn^LiKO^ mice. In female mice, however, a lower percentage of TFE3 and TFEB was found in the nuclear fraction, indicating reduced activation (Fig. 2B, C).

The loss of Flcn and the subsequent constitutive activation of TFEB and TFE3 have been associated with hyperactivation of the mTORC1 pathway, which is known to regulate cell growth and metabolism [13, 14]. Consistently with this, mTORC1 activity, as assessed by immunoblot analysis for the phosphorylated 4EBP1 protein (P-4EBP1), a direct target of mTORC1 [27], was significantly increased in male Flcn^LiKO^ mice compared to controls (Fig. 2A). However, no change in mTORC1 activation was observed in female Flcn^LiKO^ mice (Fig. 2A).

We next sought to further assess the differences between male and female mice in response to liver injury and recovery. To this end, we subjected mice to an established protocol of acute liver injury by administering a 100 mg/kg dose of diethyl nitrosamine (DEN), which is known to induce DNA damage, cell death responses, and compensatory proliferation in the liver [28]. Control mice exhibited typical hepatocyte damage at 24 hours, with complete recovery by 48 h post-treatment (Fig. S4A). In contrast, both male and female Flcn^LiKO^ mice showed more extensive liver damage, with larger areas of ballooning hepatocytes observed at 24 and 48 h post-injection (Fig. S4A). Interestingly, immunostaining for TFE3 and TFEB in liver samples at 48 h post-injection revealed similar levels of activation in both male and female Flcn^LiKO^ mice (Fig. S4B). These results suggest that both genders exhibit a comparable response to acute liver injury, with persistent damage and altered homeostasis in Flcn^LiKO^ mice.

Taken together, these findings suggest that the observed differences in liver damage between male and female Flcn^LiKO^ mice are due to gender-specific differential activation of TFE3 and TFEB in basal conditions, with male mice exhibiting stronger activation of both factors, leading to increased mTORC1 signaling and more pronounced liver injury.

### Loss of hepatic FLCN induces spontaneous liver cancer

**Fig. 3:**
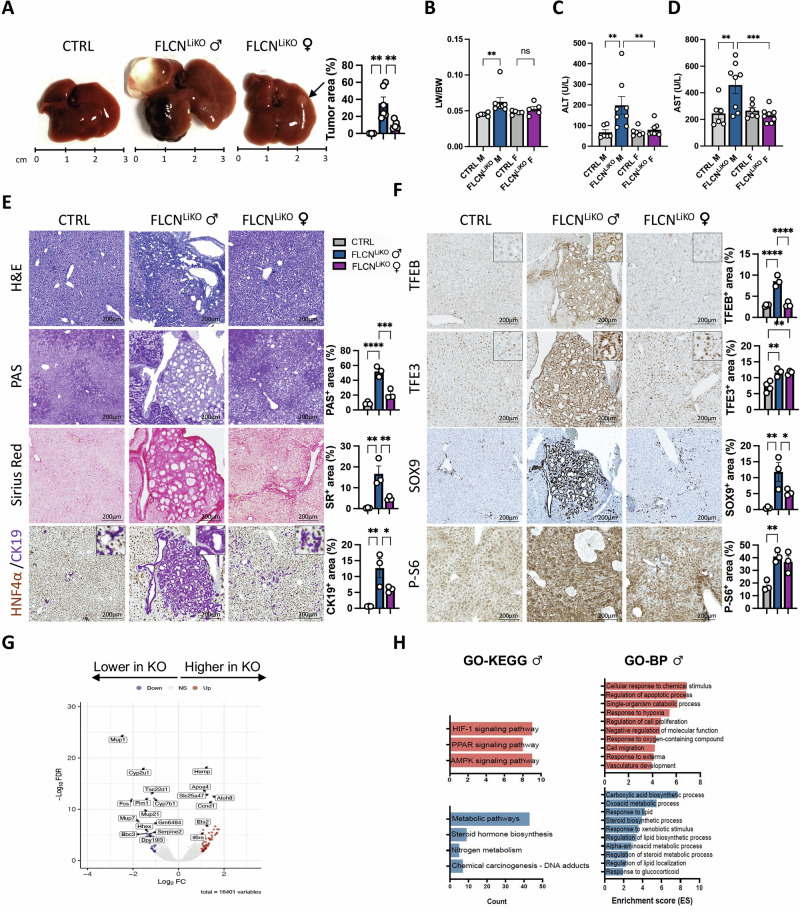
Spontaneous development of liver tumors in Flcn^LiKO^ mice. **A**–**D**. Gross liver appearance and tumor area quantification (n = 6 Flcn^LiKO^ males and n = 5 Flcn^LiKO^ females) (**A**), liver-to-body weight (LW/BW) ratio (**B**), serum ALT (**C**) and AST (**D**) levels in 90-week-old male and female Flcn^LiKO^ mice and controls (n = 6 CTRL and n = 8 Flcn^LiKO^). Tumor area is represented as percentage of the total liver area. The arrow in (**A**) indicates liver tumor. **E**, **F**. Histological characterization of liver pathology in 90-week-old Flcn^LiKO^ mice assessed by H&E, PAS, and Sirius red staining, as well as immunostainings for the indicated markers with relative quantifications (n = 3 per group). **G** Volcano plot of differentially expressed genes (DEGs) between 90-week-old male Flcn^LiKO^ and control mice. **H** Gene Ontology (GO) analysis, including KEGG and Biological Process terms, highlighting significantly upregulated (red) and downregulated (blue) pathways in Flcn^LiKO^ mice compared to control (FDR < 0.05). Each dot represents an individual mouse. Data are mean ± standard error. Statistical analysis: One-way ANOVA: *p-value < 0.05; **p-value < 0.01; ***p-value < 0.001; ****p-value < 0.0001.

Consistent with the chronic liver injury observed in young Flcn^LiKO^ mice, we found that these mice develop liver tumor and cysts by 90 weeks of age (Fig. 3A). This was associated with an increased liver-to-body weight (LW/BW) ratio (Fig. 3B), and elevated serum levels of alanine aminotransferase (ALT) (Fig. 3C), and aspartate aminotransferase (AST) (Fig. 3D). While no tumors were detected in the Flcn^fl/fl^ control mice, 100% of male and 30% of female Flcn^LiKO^ mice developed cysts and solid tumors. Notably, liver tumors and liver toxicity were significantly more pronounced in male Flcn^LiKO^ mice than in females (Fig. 3A–D).

Liver histology revealed the presence of hyperplasia of bile ducts, altered morphology and increased size and number of cholangiocytes in male Flcn^LiKO^ mice (Fig. 3E). These features resembled the cholangiocarcinoma (CCA)-like phenotype observed in mice overexpressing TFEB (TFEB^OE^) in the liver [11]. This phenotype was associated with glycogen and collagen deposition, as shown by PAS and Sirius Red staining (Fig. 3E), as well as lipid accumulation, indicated by oil-Red-O staining (Fig. S5A). In contrast, female Flcn^LiKO^ mice exhibited increased steatosis and fibrosis, but without significant alterations in biliary cell morphology (Figs. 3E and S5A). Immunostaining for CK19, a cholangiocyte marker, revealed cysts and packed cholangiocytes in male Flcn^LiKO^ mice, while female mice displayed a ductular reaction (Fig. 3E). The regions showing neoplasm of the bile ducts showed nuclear TFEB, TFE3, and SOX9, and hyperactivation of mTORC1 as evidenced by immunostaining for the phosphorylated S6 protein of the 40S ribosomal subunit (P-S6), used as readout of mTORC1 activity [27, 29, 30] (Fig. 3F).

Transcriptomic analysis revealed 443 DEGs between Flcn^LiKO^ and control male mice (Fig. 3G) and 255 in female mice (Fig. S5B). GOEA and KEGG pathway analysis performed on DEGs in the dataset from the aged male Flcn^LiKO^ mice showed a significant inhibition of several metabolic pathways such as carboxylic acid metabolic processes, normally activated in response to liver damage, and lipid biosynthesis, as previously reported in Flcn-depleted livers [22] (Fig. 3H and Table S5). In line with the observed phenotype, upregulated pathways were mostly associated with tumor development and progression, including response to hypoxia, cell proliferation, and cell migration. Conversely, GOEA and KEGG pathway analysis on DEGs in females showed inhibition of pathways associated with defense response and cell migration, and induction of organic metabolic processes and response to endogenous stimulus (Fig. S5C and Table S6). Notably, only 49 genes were in common between males and females, of which the 28 most upregulated genes were mostly involved in cell cycle regulation, such as *Ccnd1*, *Nupr1*, and *Mapre3* (Fig. S5D and Table S7), further confirming the gender differences observed in Flcn^LiKO^ mice. GOEA and KEGG pathway analysis on differentially dysregulated transcripts in males and females are reported in Table S8.

Taken together, these findings underscore the role of FLCN as a tumor suppressor in the liver, particularly in male mice, where the loss of FLCN leads to a more aggressive tumor phenotype. Moreover, the observed sexual dimorphism highlights the distinct molecular pathways underlying liver tumor development in males and females.

### Loss of *Flcn* in the liver enhances the susceptibility to DEN- and DDC-induced hepatocarcinogenesis

**Fig. 4:**
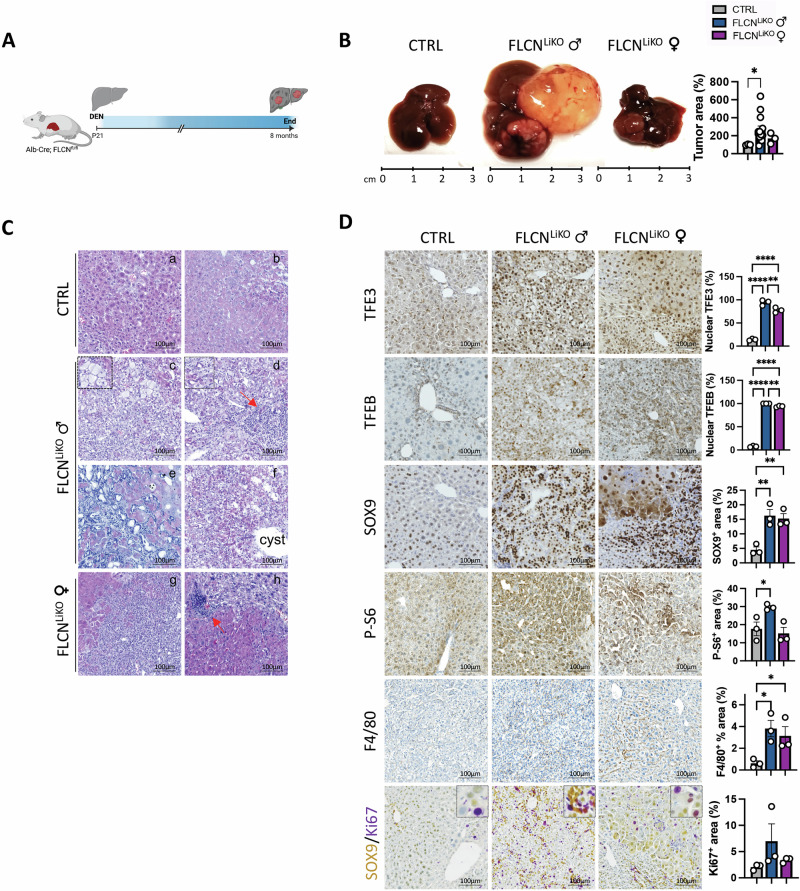
DEN-induced tumorigenesis due to Flcn depletion. **A** Schematic representation of the experimental plan. **B** Gross liver morphology and tumor area quantification in Flcn^LiKO^ and control mice injected with DEN at P21 and collected 8 months post-injection. Tumor area is represented as a percentage relative to controls (n = 3 CTRL, n = 14 male Flcn^LiKO^ mice, n = 3 female Flcn^LiKO^ mice). **C**, **D** H&E staining (**C**) and immunostainings for the indicated markers with relative quantifications (n = 3 per group) (**D**) in liver sections from Flcn^LiKO^ and control mice injected with DEN at P21 and collected 8 months post-injection. H&E staining reveals normal histological architecture in control mice (a,b), whereas Flcn^LiKO^ mice exhibit various histological subtypes of hepatocellular carcinoma (HCC) and cholangiocarcinoma (CCA), including clear cells (c), lobular and trabecular formation (d), biliary reaction (e) cyst formation (f) and immune infiltrates (d,g,h). Red arrows indicate immune infiltrates. Each dot represents an individual mouse. Data are mean ± standard error. Statistical analysis: One-way ANOVA: *p-value < 0.05; **p-value < 0.01; ***p-value < 0.001; ****p-value < 0.0001.

To further assess the role of FLCN in hepatocarcinogenesis, we induced liver injury and recovery by administering a 25 mg/kg dose of diethylnitrosamine (DEN) in Flcn^LiKO^ and control mice at post-natal stage (P) 21 (Fig. 4A), a well-established protocol for liver cancer induction [28, 31, 32]. At 8 months of age, all analyzed Flcn^LiKO^ mice, both male and female, developed solid tumors and cysts of varying sizes in the liver, whereas only 30% of control mice developed tumors (Fig. 4B), consistent with the results obtained in 90-week-old mice. H&E staining of liver sections revealed heterogeneity among the tumors, with extensive hepatocyte ballooning, cytoplasmic vacuolization (clear cells), lobular and trabecular formation, inflammatory cell infiltration, and bile duct proliferation- hallmarks of hepatocellular carcinoma (HCC) and cholangiocarcinoma (CCA)- following DEN treatment (Fig. 4C). In line with our previous findings, tumor areas showed strong nuclear staining for TFE3, TFEB, and SOX9 (Fig. 4D). As expected, mTORC1 activity, as indicated by immunostaining for P-S6, inflammation, as demonstrated by immunostaining for F4/80, and proliferation of both hepatocytes and SOX9^+^ cells, indicated by Ki67 immunostaining, were increased in male Flcn^LiKO^ mice (Fig. 4D).

**Fig. 5:**
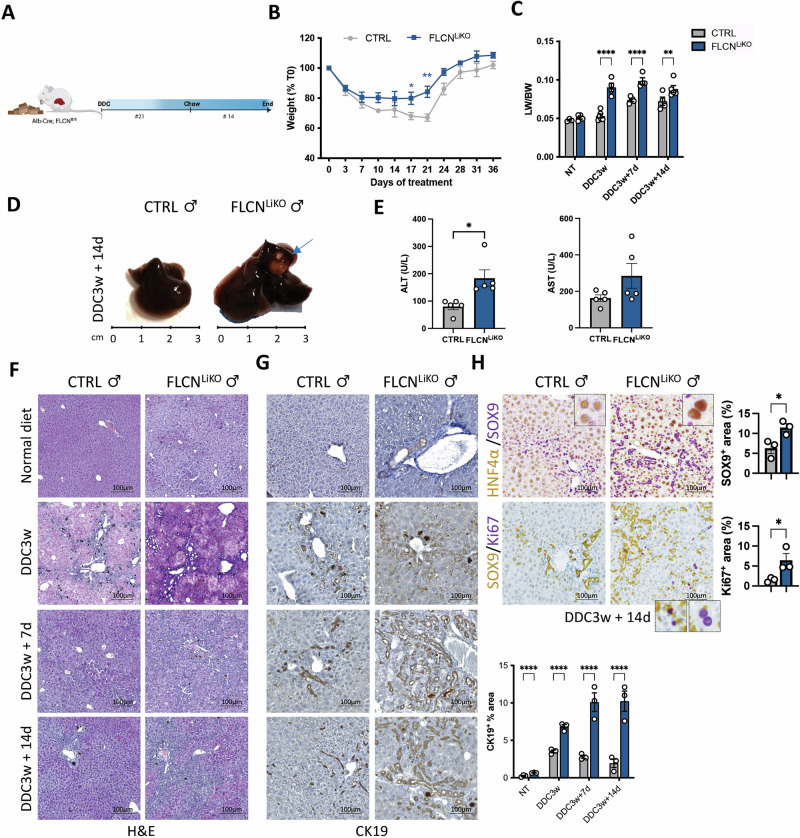
Impaired cell homeostasis due to FLCN loss during chronic liver damage and recovery. **A** Schematic representation of the experimental plan. **B** Change in body weight of Flcn^LiKO^ and control mice during the injury protocol (n = 5 per group). **C** Liver-to-body weight (LW/BW) ratio of Flcn^LiKO^ and control mice at the indicated time points during liver injury and recovery (n = 4-5 per group). **D**, **E** Gross liver appearance (**D**) and serum ALT and AST levels (**E**) in Flcn^LiKO^ and control mice 2 weeks after removal of DDC-containing food (n = 5 per group). The blue arrow indicates a liver cyst. **F**–**H** H&E staining (**F**) and immunostaining for the indicated markers (**G**, **H**) in liver sections from Flcn^LiKO^ and control mice at the specified time points, with relative quantification (n = 3 per group). All the data refer to male mice. Each dot represents an individual mouse. Data are mean ± standard error. Statistical analysis: Two-way ANOVA or Student *t*-test: *p-value < 0.05; ****p-value < 0.0001.

Next, we subjected Flcn^LiKO^ and aged-matched control mice to a well-established protocol of liver injury induced by 0.1% 3,5-diethoxycarbonyl-1,4-dihydrocolidine (DDC)-containing food followed by 2 weeks of recovery (Fig. 5A). This protocol induces hepatocyte damage and triggers a strong ductular reaction that resolves in 14-21 days in wild-type (WT) mice. However, dysregulated cell homeostasis and differentiation can lead to unresolved biliary reactions and cancer development as we previously reported in TFEB^OE^ mice [11]. As expected, control mice lost almost 20-30% of initial body weight during DDC treatment, while recovering body weight after a return to normal food (Fig. 5B). Interestingly, Flcn^LiKO^ mice were more resistant to weight loss and gained more weight than control mice during recovery (Fig. 5B), likely as a consequence of the initial protection to liver injury due to constitutive activation of TFEB and TFE3, as previously reported for different protocols of chronic damage [21, 22]. The liver-to-body weight (LW/BW) ratio was significantly higher in Flcn^LiKO^ mice compared to controls during the injury protocol (Fig. 5C, D). Moreover, serum ALT and AST levels were significantly increased in Flcn^LiKO^ mice (Fig. 5E), indicating impaired cell homeostasis.

Histological analysis (H&E and CK19 immunostaining) revealed increased liver damage and ductal reaction in Flcn^LiKO^ livers after 3 weeks on the DDC diet (Fig. 5F, G). Co-immunostaining for HNF4α and SOX9 highlighted the presence of hybrid hepatocytes and progenitor cells, and increased hepatocyte proliferation (Fig. 5H).

In contrast to the strong difference observed between males and females in DEN-induced liver cancer and in the development of spontaneous tumors, both male and female Flcn^LiKO^ mice exhibited similar responses to DDC-induced injury and recovery, including increased weight gain (Fig. S6A), and LW/BW ratio (Fig. S6B), higher serum ALT and AST levels (Fig. S6C), impaired biliary regeneration (Fig. S6D), ductular reaction, as demonstrated by SOX9 immunostaining (Fig. S6E), and abnormal cell differentiation, as demonstrated by HNF4α and SOX9 co-immunostaining (Fig. S6E). Both sexes also showed increased hepatocyte proliferation (Fig. S6E), suggesting a comparable response to chronic liver damage and regeneration. The similar response of male and female Flcn^LiKO^ mice to DDC-induced liver injury and recovery likely reflects the increased levels of TFE3, and to a less extent of TFEB, in Flcn^LiKO^ mice compared to controls (Fig. S6F).

Since Alb-Cre recombination leads to deletion of *Flcn* in both hepatocytes and cholangiocytes [11], we generated mice with hepatocyte-specific deletion of *Flcn* to investigate the effect in these cells. We injected Flcn^fl/fl^ mice with an adeno-associated virus serotype 8 (AAV8) expressing Cre (Flcn^HepKO^), or GFP as control, under the hepatocyte-specific thyroxin-binding globulin promoter (TBG) (Fig. S7A). This resulted in nearly 99% recombination in hepatocytes [11] and a concomitant reduction of more than 90% in Flcn expression (Fig. S7B). In line with the results obtained in Flcn^LiKO^ mice, Flcn depletion in hepatocyte of adult mice resulted in strong nuclear localization of TFE3 and TFEB, with almost 100% of hepatocytes showing nuclear TFE3 (Fig. S7C). Under basal conditions, Flcn^HepKO^ mice did not show any significant alterations in body weight or in the LW/BW ratio (Fig. S7D). However, similarly to Flcn^LiKO^, Flcn^HepKO^ mice showed increased body weight, increase in the LW/BW ratio (Fig. S7D, E), and increased serum transaminases (Fig. S7F) in response to DDC-induced liver damage. Untreated Flcn^HepKO^ mice displayed a phenotype that looked similar to Flcn^LiKO^ mice, albeit milder, with clear hepatocytes and accumulation of glycogen (Fig. S7G). This phenotype was exacerbated after DDC-induced liver injury and recovery, showing increased hepatocyte damage, as demonstrated by cytoplasmic clearing and ductular reaction, as indicated by CK19 immunostaining (Fig. S7H).

These findings confirm that loss of hepatic Flcn increases the susceptibility to chemically induced liver tumors by disturbing liver homeostasis.

### TFE3 is the main driver of liver carcinogenesis in Flcn^LiKO^ mice

**Fig. 6:**
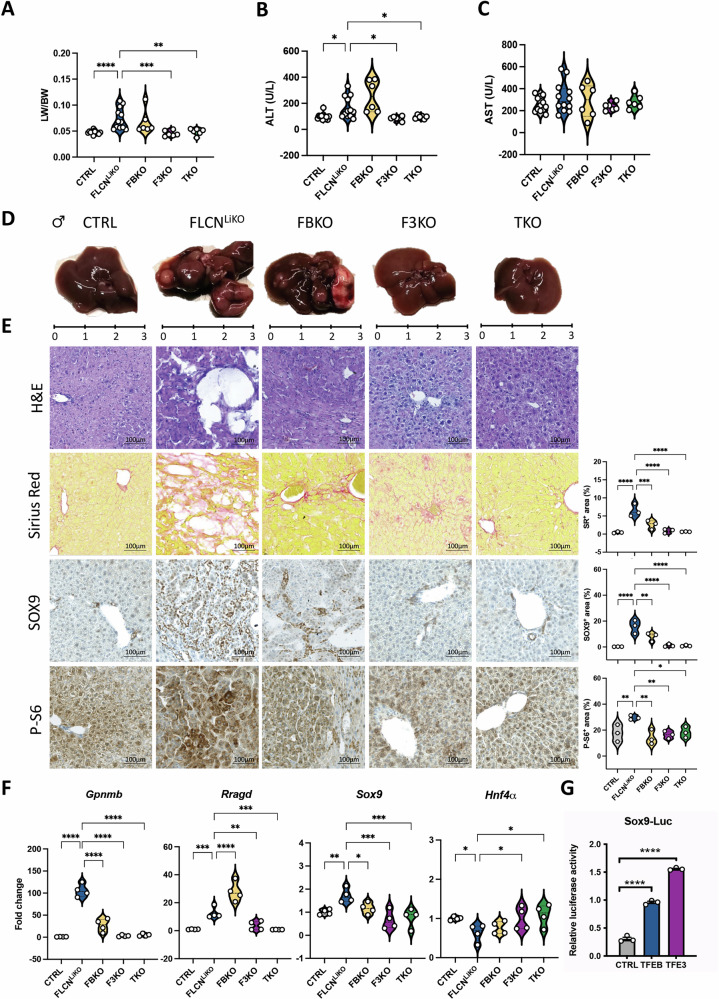
TFE3 is the principal contributor to liver pathology in Flcn^LiKO^ mice. **A**–**E** Liver-to-body weight (LW/BW) ratio (**A**) and serum ALT (**B**) and AST (**C**) levels in control, Flcn^LiKO^, FBKO, F3KO, and TKO mice 8 months after DEN injection (n = 12 CTRL and Flcn^LiKO^, n = 6 FBKO, F3KO, TKO). Gross liver morphology (**D**) and histological analysis with relative quantifications (n = 3 per group) (**E**), showing partial rescue upon TFEB depletion and complete rescue in TFE3- or TFEB/TFE3-depleted livers. **F** Gene expression analysis of the indicated genes in liver samples from mice of the indicated genotypes (n = 4 per group). **G** Luciferase assay of *Sox9* promoter activity demonstrating transactivation upon TFEB and TFE3 overexpression (n = 3 per group). All the data refer to male mice. Each dot represents an individual mouse. Data are mean ± standard error. Statistical analysis: One-way ANOVA: *p-value < 0.05; **p-value < 0.01; ***p-value < 0.001; ****p-value < 0.0001.

We next investigated to what extent TFE3 and/or TFEB are responsible for the phenotype observed in Flcn^LiKO^ mice. We generated mice lacking Flcn and either TFEB (Flcn^flox/flox^; Alb-Cre^+^; Tfeb^flox/flox^ mice, hereafter FBKO) or TFE3 (Flcn^flox/flox^; Alb-Cre^+^; Tfe3^KO^ mice, hereafter F3KO), or both (Flcn^flox/flox^; Alb-Cre^+^; Tfeb^flox/flox^; Tfe3^KO^ mice, hereafter TKO) in the liver. To assess their role in carcinogenesis in livers lacking Flcn, we injected mice with DEN at P21 and collected livers 8 months post-injection. Remarkably, we observed a complete rescue in F3KO and TKO mice with a significant reduction in LW/BW (Fig. 6A), reduction of serum ALT and AST levels (Fig. 6B, C), and tumor development (Fig. 6D). In contrast, depletion of TFEB only partially rescued the neoplastic phenotype following DEN injection. Specifically, tumors were detected in 75% of Flcn^LiKO^ mice and 50% of FBKO mice, whereas only 15% of F3KO mice and no TKO mice developed tumors. In agreement with these findings, FBKO mice still showed an increased LW/BW ratio, development of liver cysts, and elevated serum ALT and AST levels, similar to Flcn^LiKO^ mice, while F3KO and TKO mice showed marked improvements (Fig. 6A–D).

We then sought to determine if TFE3 was upregulated in FBKO as a compensatory mechanism explaining the observed phenotype. qPCR analysis confirmed the expression of *Tfe3* in FBKO and of *Tfeb* in F3KO (Fig. S8A), and immunoblot analysis showed a significant reduction in TFE3 and TFEB protein levels in FBKO and F3KO, respectively (Fig. S8B). These data suggest that the differential impact of TFE3 and TFEB depletion in Flcn^LiKO^ mice may be attributed to their distinct expression levels in the liver, as we reported in young mice (Fig. 2).

In addition, deletion of TFE3 in F3KO led to a reduction in liver fibrosis, a decrease in SOX9 expression in hepatocytes, and normalization of mTORC1 activity, as demonstrated by P-S6 immunostaining and immunoblot for P-4EBP1 (Figs. 6E and S8B). In contrast, FBKO livers showed only a partial reduction in these markers (Figs. 6E and S8B). Gene expression analysis also showed normalization of TFEB/TFE3 targets preferentially in F3KO and TKO mice (Fig. 6F), including *Sox9* that we confirmed to be a TFE3 target, as we reported previously for TFEB [11] (Fig. 6G). Interestingly, the transactivation of the *Sox9* promoter due to TFE3 overexpression was stronger compared to TFEB, suggesting that, even though TFE3 protein levels was reduced in FBKO mice, it may compensate for TFEB loss, driving the observe phenotype.

Additionally, markers of hepatocyte identity, such as *Hnf4α*, *Albumin* and *Arg1*, reduced in Flcn^LiKO^ mice due to abnormal hepatocyte differentiation, were significantly restored in TKO mice compared to FLCN^LiKO^ mice (Figs. 6F and S8C). In contrast, markers of cholangiocyte identity, including *Sox9*, *Epcam*, and *Krt19*, which were strongly upregulated in Flcn^LiKO^ mice, were fully normalized in TKO mice (Figs. 6F and S8C).

Taken together, these results confirm that constitutive activation of TFE3 plays a central role in driving enhanced tumor development in Flcn^LiKO^ livers.

## Discussion

Primitive liver cancer is a malignant tumor that originates in the liver classified as hepatocellular carcinoma (HCC) or cholangiocarcinoma (CCA), depending on the cell type of origin [2, 3]. Despite extensive studies on the pathological features of liver cancer, the mechanisms driving the malignant transformation of liver cells and promoting cancer progression remain poorly understood. Furthermore, effective therapeutic strategies are still lacking. Liver tumor development is a multistep process induced by liver damage followed by inflammation, and cycles of necrosis and regeneration [33]. This sequence of events leads to neoplastic transformation in the long term due to a more permissive environment. However, how cancer development and environmental factors are linked is poorly understood.

The mechanistic target of rapamycin complex 1 (mTORC1) is a key metabolic hub controlling cell growth and homeostasis in response to nutrients [34, 35]. mTORC1 activates anabolic pathways, including lipid and protein synthesis, and inhibits catabolic pathways like autophagy and oxidative metabolism [36]. Aberrant mTORC1 activation is a hallmark of many cancers, including HCCs [37, 38]. Both mTORC1 hyperactivation [39] and persistent inhibition [40] result in liver damage and enhanced HCC development. Interestingly, sporadic HCC development has been described in a liver-specific TSC1 knockout (Tsc1^LiKO^) mouse model characterized by chronic activation of mTORC1. In this model, cancer development was preceded by liver damage, necrosis, and regeneration, which leads to spontaneous transformation and cancer [39, 41].

FLCN, a GTPase-activating proteins (GAP) for the RagC and RagD GTPases, negatively regulates transcription factors TFEB and TFE3 by enabling their phosphorylation by mTORC1, which inhibits their cytoplasm-to-nucleus translocation [26, 42, 43]. Furthermore, TFEB/TFE3 and mTORC1 have been implicated in a feedback loop in which mTORC1 negatively regulates TFEB/TFE3, which in turn positively regulate mTORC1 activity by inducing the transcription of RagC/D [14]. Consistent with this, FLCN depletion in cells and tissues is associated with constitutive nuclear localization of TFEB and TFE3, resulting in mTORC1 hyperactivation [44]. Recent studies have shown that FLCN-mediated regulation of mTORC1 is substrate-specific and is only relevant for the phosphorylation of TFEB and TFE3, whereas it is not involved in the phosphorylation of other mTORC1 substrates such as S6 kinase beta-1 (S6K1) and eukaryotic translation initiation factor 4E–binding protein 1 (4E-BP1) [13]. This led to the identification of a non-canonical branch of the mTORC1 pathway, which has distinct properties and functions compared to the canonical branch [13, 17].

Mutations in the FLCN gene cause Birt-Hogg-Dube’ (BHD) syndrome, an inherited familiar cancer syndrome mainly characterized by skin and kidney cancer [15, 16]. An emerging, although rare, aspect of the BHD phenotype is the formation of intestinal polyps and pancreatic and liver cysts [45–47]. However, the role of FLCN in liver carcinogenesis and cystogenesis has never been explored so far. We recently reported that TFEB overexpression caused liver damage, impaired cell differentiation, and induced a CCA-like phenotype in a time- and dose-dependent manner [11]. Moreover, we described how autophagy activation, due to TFEB overexpression, plays a critical role in cilia disassembly, favoring tumor growth in CCA [12]. These observations prompted us to test whether FLCN depletion, similarly to TFEB overexpression, might impair liver homeostasis and promote carcinogenesis. We found that FLCN deficiency in the liver (Flcn^LiKO^ mice) led to hepatocellular damage, impaired recovery from both acute and chronic injury, and development of liver tumors with heterogenous histological and biochemical features. These results suggest that FLCN is a novel player in liver cancer development, bridging the gap between environmental stressors and cell adaptation.

Recent studies have shown that depletion of FLCN specifically in the mouse liver protects mice from developing fibrosis and inflammation upon exposure to a non-alcoholic steatohepatitis (NASH)-inducing diet. This effect was linked to the activation of MiT/TFE factors [21, 22], which are involved in lipid catabolism [25, 48]. While these findings suggest that activating MiT/TFE factors, through FLCN depletion, could be beneficial for NAFLD and NASH therapy, our data highlight potential long-term consequences. In our study, FLCN depletion led to liver carcinogenesis at 90 weeks of age, in contrast to previous studies that focused on much earlier stages (8-13 weeks of age) [21, 22].

Notably, consistent with TFEB^OE^ mice, our results revealed a strong biliary phenotype in Flcn^LiKO^ mice characterized by bile duct proliferation and enlargement, leading to CCA development. These findings are consistent with those observed in Tsc1^LiKO^ mice, which also exhibit mTORC1 hyperactivation and ductular reaction [49].

Interestingly, FLCN expression in primary cilia has been reported to be involved in the onset of ciliogenesis [50]. In cholangiocytes, primary cilia function as a multisensor of the extracellular milieu, detecting a wide variety of chemical and physical stimuli. Loss of primary cilia has been reported in different tumoral tissues, including CCA [51]. The effects of Flcn depletion in cilia formation in cholangiocytes and the consequences in tumor formation have never been reported. Our data suggest that Flcn plays a key role in cholangiocyte homeostasis. However, further studies are required to fully understand the mechanism involved.

An intriguing finding in our study was the pronounced sexual dimorphism in tumor development in Flcn^LiKO^ mice. Males exhibited a much more severe phenotype, with a higher frequency and larger tumors compared to females. Sexual dimorphism has been described in several liver pathologies, including HCC [52], although the underlying mechanisms remain unclear. We demonstrated that male mice have higher levels of TFEB and TFE3 expression, as well as more prominent nuclear translocation of these transcription factors, which may contribute to the more severe phenotype observed in males. We speculate that the observed differences in expression levels and nuclear localization between untreated male and female Flcn^LiKO^ mice may be due to hormonal regulation of TFE3 and TFEB activation. However, further studies are needed.

Our data pinpoint TFE3 as the principal player in the liver phenotype observed in Flcn^LiKO^ mice and highlights the role of TFE3 in the regulation of SOX9 expression, as we previously reported for TFEB. Interestingly, TFE3 had a stronger effect on the SOX9 promoter compared to TFEB, suggesting that TFE3 may compensate for TFEB loss in FBKO mice, partially rescuing the phenotype. These observations are in line with our previous work in kidney-specific Flcn knockout mice, where TFEB deletion rescued the cystogenic phenotype, suggesting functional redundancy and cooperation between TFEB and TFE3 [13]. However, using a mouse xenograft generated using a cell line derived from a renal cancer patient with BHD syndrome, we found that silencing of either TFEB or TFE3 was able to rescue the tumorigenic phenotype [13, 17]. Nevertheless, in our liver model, deletion of TFE3, but not TFEB, completely rescued the liver phenotype, underscoring the dominant role of TFE3 in this context. These differences may stem from the distinct expression levels of TFEB and TFE3 in hepatocytes, with TFE3 expression being notably higher than TFEB in both control and Flcn^LiKO^ mice [17].

Neither HCC nor CCA have been reported in BHD patients so far. A correlation between FLCN mutations and liver cyst development has been reported but remains controversial. It is important to point out that BHD patients are germline heterozygous for loss-of-function mutations of the FLCN gene. However, several aspects of BHD syndrome phenotypes, including renal cystic and tumorigenic phenotypes, depend on somatic second hit mutations in the FLCN gene, leading to loss-of-heterozygosity. This appears to be a frequent event in the kidney, whereas it is rare in the liver. It is possible that this difference is related to the polyploid nature of hepatocytes [53], making loss-of-heterozygosity more difficult to achieve. Further studies are needed to better understand these differences and their implications for liver carcinogenesis.

Altogether, our study unveils a novel role for FLCN in liver homeostasis and as a tumor suppressor in liver carcinogenesis.

## Material and methods

### Mouse experiments

All mice had a C57BL/6J strain background. Standard food and water were given *ad libitum*. Flcn^flox/flox^, Tfe3^KO^, and Tfeb^flox/flox^ mice were described previously [9, 26, 54]. Wild-type and *Albumin*-Cre mouse lines were obtained from the Jackson laboratory (Bar, Harbor, ME). The liver-specific Flcn and Tfeb knock-out mouse lines were generated by crossing the Flcn^flox/flox^ and Tcfeb^flox/flox^ mouse lines with *Alb-*Cre transgenic mice.

To induce liver injury, mice were injected intraperitonially with DEN 100 mg/Kg (at 12 weeks of age) or 25 mg/kg (at P21) in corn oil, and livers were collected 24-, 48-, or 8-months post injection for analysis. For the chronic liver injury protocol, mice fed 0.1% DDC food (Custom Animal Diet, Bangor, PA), as indicated in the text. After injury, mice were provided with a normal chow diet and drinking water.

Power analysis was used to calculate the sample size required for animal experiments and animals were randomized to the different groups. Animals were assigned to the experimental groups based on the genotype and the data randomly collected and processed. There is no blinding of researchers or participants.

### AAV generation and injection

The pAAV-TBG-CRE vector was described previously [11]. The pAAV-TBG-GFP vector was provided by the AAV TIGEM Vector Core. The AAV8 viral vectors were produced by Innovavector as previously described [55]. Injections of AAV8-TBG-GFP and AAV8-TBG-CRE were performed in a volume of 200 μL at a dose of 5 × 10^12^ GC/kg of body weight in the retroorbital plexus of 6-week-old Flcn^flox/flox^ mice. Mice were sacrificed at 12 weeks post injection or subjected to the DDC injury protocol as described above, and liver samples were harvested for analyses.

### Cell culture, plasmids, and transfection reagents

HEK293 cells were cultured in Dulbecco’s Modified Eagle Medium with 10% fetal bovine serum, 5% penicillin/streptomycin, and 2 mM L-glutamine. Cells were tested for mycoplasma contamination. For luciferase assays, the pGL4-Sox9 promoter plasmid was previously described [11]. The Sox9 promoter-reporter luciferase construct was transfected along with pRL-TK (Promega, Madison, MI) in HEK293 cells using lipofectamine LTX following the manufacturer’s instructions. Twenty-four hours after transfection, the cells were collected and subjected to luminescence detection using the Dual-Luciferase Reporter Assay system (Promega, Madison, MI). The luminescence was measured using a Glomax Luminometer (Promega, Madison, MI) and normalized against Renilla luciferase activity.

### Analysis of serum samples

Blood samples were collected by retroorbital bleeding. Serum was frozen at −80 °C or used immediately after collection. ALT and AST were measured using the VitroVet system (Scil) following the manufacturer’s protocol (Medline) for ALT (CCA00026) and AST (CCA00025).

### RNA extraction and quantitative RT-PCR

Total RNA was extracted in QIAzol lysis reagent (Qiagen, Hilden, Germany) using a RNeasy kit (Qiagen, Hilden, Germany). RNA was reverse transcribed using a first strand complementary deoxyribonucleic acid kit with random primers according to the manufacturer’s protocol (Applied Biosystems). The RT-PCR reactions were performed using the Roche Light Cycler 480 system (Roche). The PCR reaction was performed using SYBR Green Master Mix (Roche) with the following thermocycler conditions: pre-heating, 5 min at 95 °C; cycling, 40 cycles of 15 s at 95 °C, 15 s at 60 °C and 25 s at 72 °C. Results were expressed in terms of cycle threshold (C_t_). The C_t_ values were averaged for each duplicate. The *Ribosomal protein S16* gene was used as an endogenous control (reference marker). Differences between the mean C_t_ values of the tested genes and those of the reference gene were calculated as ∆C_tgene_ = C_tgene_ − C_treference_. Relative fold increase in expression levels is given as 2^−∆∆Ct^. Primers used for RT-PCR are listed in Table S9.

### Immunoblot analysis

Liver samples were homogenized in RIPA buffer (50 mM Tris–HCl pH 7.4, 150 mM NaCl, 1% Triton X-100, 1 mM EDTA pH 8.0, 0.1% SDS) containing complete protease inhibitor cocktail (Roche Diagnostics, Indianapolis, IN). Samples were incubated for 20 min at 4 °C and centrifuged at 16,000 × *g* for 10 min. The pellet was discarded, and cell lysates were used for Western blot analysis. Ten to twenty micrograms of protein were run on a 4–20% SDS-PAGE polyacrylamide gel by electrophoresis. After transfer to a PVDF membrane, the blots were blocked in TBS-Tween 20 containing 5% non-fat milk for 1 h at room temperature, then exposed to primary antibody was applied overnight at 4 °C. Anti-rabbit IgG or anti-mouse IgG conjugated with horseradish peroxidase (GE Healthcare, Little Chalfont, UK) and ECL (Pierce, Thermo Fisher Scientific, Wilmington, DE) was used for detection. Antibodies used for immuno-blots are listed in Table S10.

### Nuclear/cytosolic fractionation

Enriched nuclear and cytosolic cellular subfractions were isolated by differential centrifugation, as previously described [48]. Briefly, the liver was minced on ice and homogenized using a Teflon pestle and mortar and suspended in mitochondrial isolation buffer (MIB; 250 mM sucrose, 20 mM HEPES, 10 mM KCl, 1.5 mM MgCl2, 1 mM EDTA, 1 mM EGTA) supplemented with protease and phosphatase inhibitor cocktails (Complete and PhosSTOP Roche, Roche Diagnostics, Basel, Switzerland). The homogenates were then centrifuged at 1000 × *g* for 10 min at 4 °C to pellet the nuclei while mitochondrial and cytosolic fractions were contained within the supernatant. The supernatant fraction was re-centrifuged twice at 16,000 × *g* for 20 min at 4 °C to pellet the mitochondria and supernatant containing cytosolic subfraction was collected. Pellets containing nuclei were re-suspended in nuclear lysis buffer (1.5 mM MgCl_2_, 0.2 mM EDTA, 20 mM HEPES, 0.5 M NaCl, 20% glycerol, 1% Triton X-100), incubated on ice for 30 min, and then sonicated 3 Å ~10 s followed by a final centrifugation step of 15 min at 16,000 × *g*. The supernatant was collected to obtain the enriched nuclear fraction. Fraction purity was determined by Western blot analysis.

### Liver staining

Liver specimens were fixed in 4% Paraformaldehyde for 12 h and stored in 70% ethanol, embedded into paraffin blocks, and cut into 6μm sections. H&E staining was performed following the IHC World protocol. For PAS-D staining, sections were rehydrated and stained with PAS-D reagent according to the manufacturer’s instructions (Bio-Optica). Sirius red staining was performed on liver sections stained for 1 h in a picro-Sirius red solution (0.1% Sirius red in a saturated aqueous solution of picric acid). After two changes of acidified water (5 mL glacial acetic acid in 1 L water), sections were dehydrated in three changes of 100% ethanol, cleared in xylene, and mounted in a resinous medium. Oil-Red-O staining was performed on 6μm sections from OCT-embedded tissues, fixed in formalin and stained following the IHC World protocol. Immunohistochemistry analyses were performed on 6µm paraffin sections with a VENTANA BenchMark Ultra automated staining instrument (Ventana Medical Systems, Roche), using VENTANA reagents except as noted, according to the manufacturer’s instructions. Slides were deparaffinized using Discovery wash buffer (cat # 950-510) for 16 min at 72 °C. Epitope retrieval was accomplished with Discovery CC1 solution (cat # 950-500) at a high temperature (95 °C) for a period that is suitable for a specific tissue type. Antibodies were titered with a blocking solution into user fillable dispensers for use on the automated stainer. Slides were developed using the Discovery ChromoMap DAB kit (cat #760-159) and Discovery Purple kit (Cat#760-229) according to the manufacturer’s instructions. Slides were then counterstained with hematoxylin II (cat # 790-2208) for 8 min, followed by Bluing reagent (cat # 760-2037) for 4 minutes. Stained liver sections were examined under a Zeiss Axiocam MR microscope or with ZEISS Axio Scan.Z1. Antibodies are listed in Table S10.

Quantifications were performed using ImageJ or Qpath software. For each study, 3 images were quantified for each liver section and n = 3 mice per genotype/group as indicated in figure legends.

### Bioinformatics workflow and functional analysis

The raw data were analyzed by the Next Generation Diagnostics srl proprietary NEGEDIA Digital mRNA-seq pipeline (v2.0). This involves a cleaning step by quality filtering and trimming, alignment to the reference genome (mm10), and counting by gene (BBMap – Bushnell B. –sourceforge.net/projects/bbmap/) [56, 57]. The raw expression data were normalized, analyzed by the NEGEDIA DEGs analysis pipeline (v1.2.0) [58] (OnRamp BioInformatics, Inc., https://cran.r-project.org/web/packages/fpc/index.html). The threshold for statistical significance of gene expression analysis was False Discovery Rate (FDR) < 0.05 (Tables S1, S2, S5 and S6). GOEA was performed separately for induced and inhibited genes for each comparison of interest using the DAVID Bioinformatic tool [59, 60] restricting the output to Biological Process (BP) and Cellular Component (CC) terms. KEGG Pathway [61] analysis was also performed. The threshold for statistical significance of GOEA was FDR < 0.1 and Enrichment Score (ES) ≥ 1.5, while for the KEGG Pathway analysis used FDR < 0.1. The differentially expressed genes identified in Flcn^LiKO^ mice *vs* control mice at 12 and 90 weeks of age and in males and females were compared, visualized by Venn diagrams (Tables S3 and S7), and analyzed by GOEA and KEGG Pathway analysis (Tables S4 and S8).

### Statistical analyses

Obtained data were processed in Excel (Microsoft Corporation) and Prism (GraphPad Software) to generate bar charts and perform statistical analyses. Data are expressed as mean values ± standard error (SE). Statistical significance was computed using Student’s two-tail t-test or ANOVA. A *p*-value < 0.05 was considered statistically significant. In all Figs., *p < 0.05, **p < 0.01, ***p < 0.001 and ****p < 0.0001.

### Data visualization

Venn diagrams and Volcan plots were generated using custom annotation scripts.

## Supplementary information

Supplementary information

## Data Availability

Transcriptomics data have been deposited in the Gene Expression Omnibus (GEO) under accession codes GSE269752 [62]. The title is “Transcriptomic analysis of Flcn^LiKO^, a study performed on males and females at 12- and 90- weeks of age”. Source data and quantifications given in the main text have associated raw data. All other data supporting the findings of this study are available from the corresponding authors on reasonable request.
