# Supplementary material for: Folliculin depletion results in liver cell damage and cholangiocarcinoma through MiT/TFE activation
Source: Cell Death Differ. 2025 Apr 6;32(8):1460–72. doi: 10.1038/s41418-025-01486-8 (PMC12325662; doi:10.1038/s41418-025-01486-8)

# Folliculin depletion results in liver cell damage and cholangiocarcinoma through MiT/TFE activation

Bruno Maria Custode[1#], Francesco Annunziata[1#], Felipe Dos Santos Matos[1], Valentina Schiano[1], Veronica Maffia[1], Milena Lillo[1], Rita Colonna[1], Rossella De Cegli[1], Andrea Ballabio[1,2,3,4] and Nunzia Pastore[1,2*]

**Table of contents**

Supplementary Figures……………………………………………………………………..8

Supplementary Tables……………………………………………….................................... 9

**Supplementary Figures**

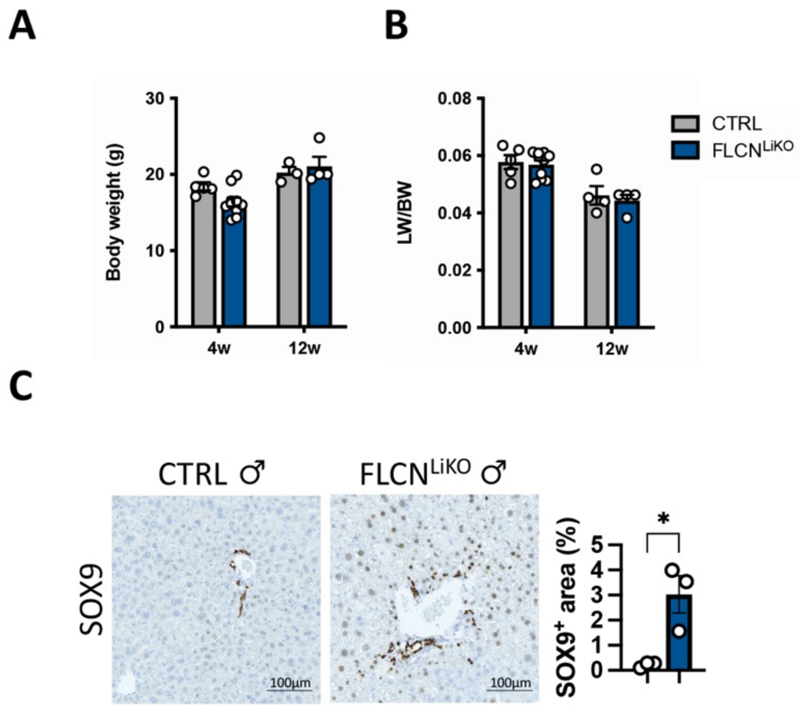


**Fig. S1. Characterization of Flcn$^{LiKO}$ male mice (referred to Fig.1)**

**A,B.** Body weight (BW) (**B**) and liver-to-body weight (LW/BW) ratio (**C**) of Flcn$^{LiKO}$ and control male mice at the indicated time points. **C.** Immunostaining for SOX9 with relative quantification (n=3 per group) in liver sections from Flcn$^{LiKO}$ and control male mice. Each dot represents an individual mouse. Data are mean ± standard error. Statistical analysis: Student *t*-test: **p*-value < 0.05.

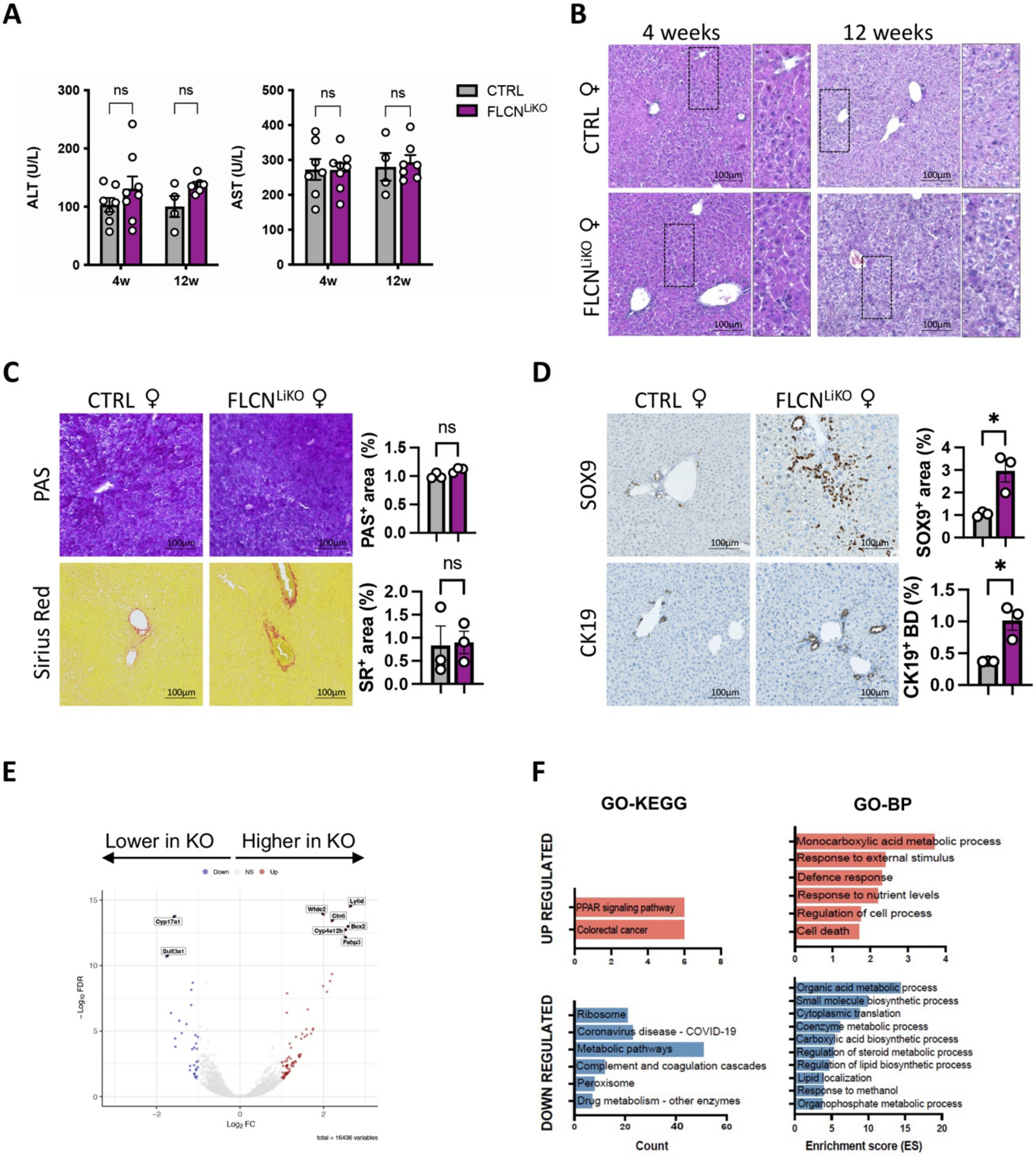

A
B
C
D
E
F
ALT (U/L)
AST (U/L)
ns
4w
12w
CTRL
FLCN^LiKO
4 weeks
12 weeks
CTRL ♀
FLCN^LiKO ♀
100µm
PAS
Sirius Red
PAS⁺ area (%)
SR⁺ area (%)
SOX9
CK19
SOX9⁺ area (%)
CK19⁺ BD (%)
*
Lower in KO
Higher in KO
Down
NS
Up
Cyp17a1
Sult3a1
Wfdc2
Ly6d
Cln6
Bex2
Cyp4a12b
Fabp3
−Log10 FDR
Log2 FC
total = 16436 variables
GO-KEGG
GO-BP
UP REGULATED
DOWN REGULATED
PPAR signaling pathway
Colorectal cancer
Monocarboxylic acid metabolic process
Response to external stimulus
Defence response
Response to nutrient levels
Regulation of cell process
Cell death
Ribosome
Coronavirus disease - COVID-19
Metabolic pathways
Complement and coagulation cascades
Peroxisome
Drug metabolism - other enzymes
Organic acid metabolic process
Small molecule biosynthetic process
Cytoplasmic translation
Coenzyme metabolic process
Carboxylic acid biosynthetic process
Regulation of steroid metabolic process
Regulation of lipid biosynthetic process
Lipid localization
Response to methanol
Organophosphate metabolic process
Count
Enrichment score (ES)

**Fig. S2. Flcn$^{LiKO}$ female mice display a milder liver pathology**

**A.** Serum ALT and AST levels of Flcn$^{LiKO}$ and control female mice at 4- and 12- weeks of age (n=7 CTRL, n=8 Flcn$^{LiKO}$ at 4weeks; n=4 CTRL, n=7 Flcn$^{LiKO}$ at 12weeks). **B.** H&E staining of liver sections from Flcn$^{LiKO}$ and control female mice at the indicated ages. **C.** Sirius Red and PAS staining of liver sections from female mice of the indicated genotypes at 12 weeks of age, with relative quantifications (n=3 per group). **D.** Immunostaining for the indicated markers on liver sections from 12-week-old Flcn$^{LiKO}$ and control female mice, with relative quantifications (n=3 per group). **E.** Volcano plot of the differentially expressed genes (DEGs) between 12-week-old Flcn$^{LiKO}$ and control female mice. **F.** Gene Ontology (GO) analysis, including KEGG and Biological Process terms, showing significantly upregulated (red) and downregulated (blue) pathways in Flcn$^{LiKO}$ female mice compared to control (FDR < 0.05). All data refer to female mice. Each dot represents an individual mouse. Statistical analysis: Data are mean ± standard error. Student *t*-test: **p*-value < 0.05.

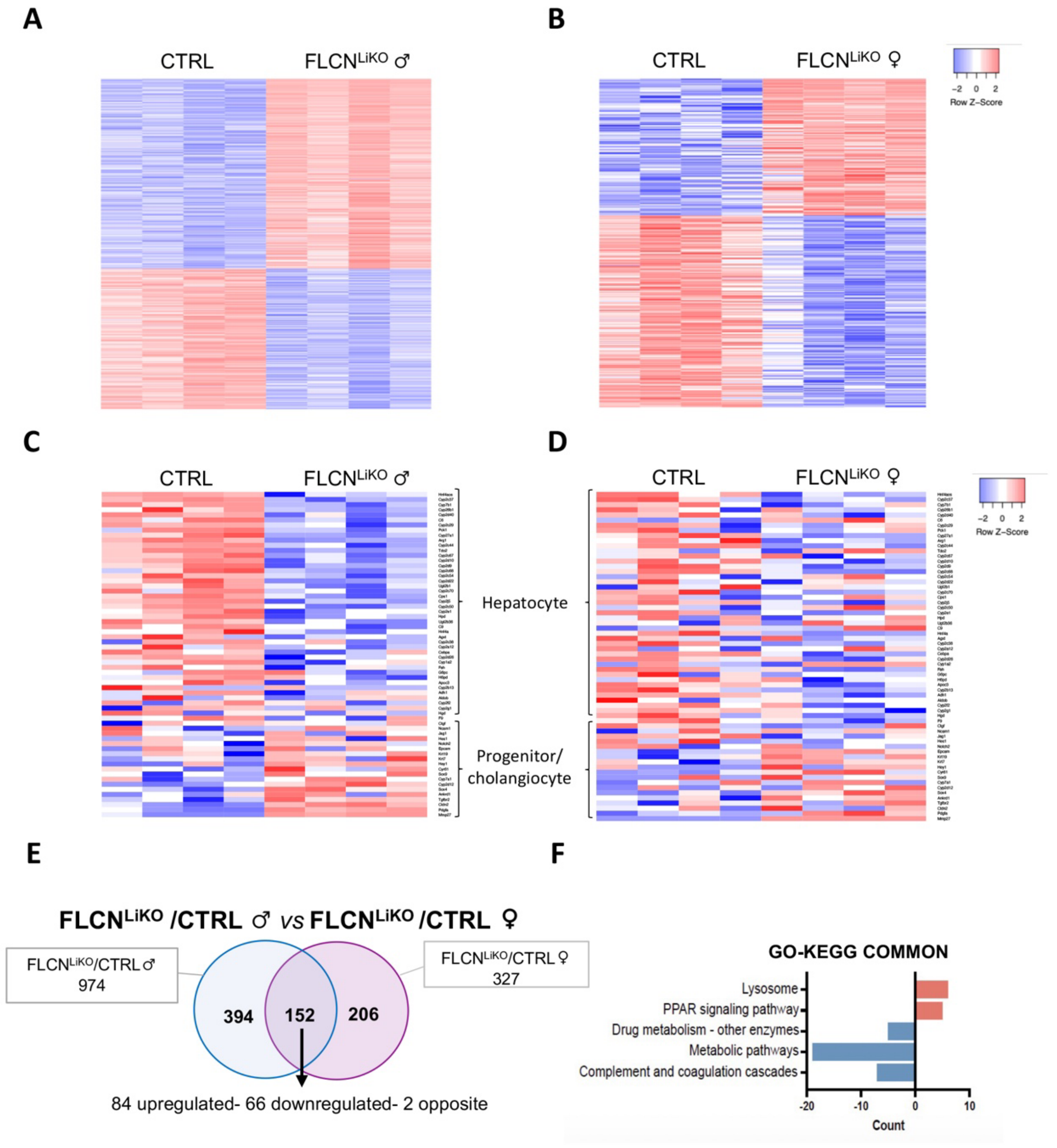


**Fig. S3. Sex-specific effects of Flcn loss on gene expression in mice**

**A,B.** Heatmap showing differentially expressed genes (DEGs) between Flcn$^{LiKO}$ and control mice in male (**A**) and female (**B**) liver. **C,D.** Heatmap showing different expression of hepatocyte-, progenitor- and cholangiocyte- specific markers between Flcn$^{LiKO}$ and control mice in male (**C**) and female (**D**) liver. **E,F.** Venn diagram comparing transcriptomic datasets in Flcn$^{LiKO}$ male and

female mice with their respective controls (**E**) and KEGG pathway analysis of the commonly upregulated or downregulated transcripts (**F**).

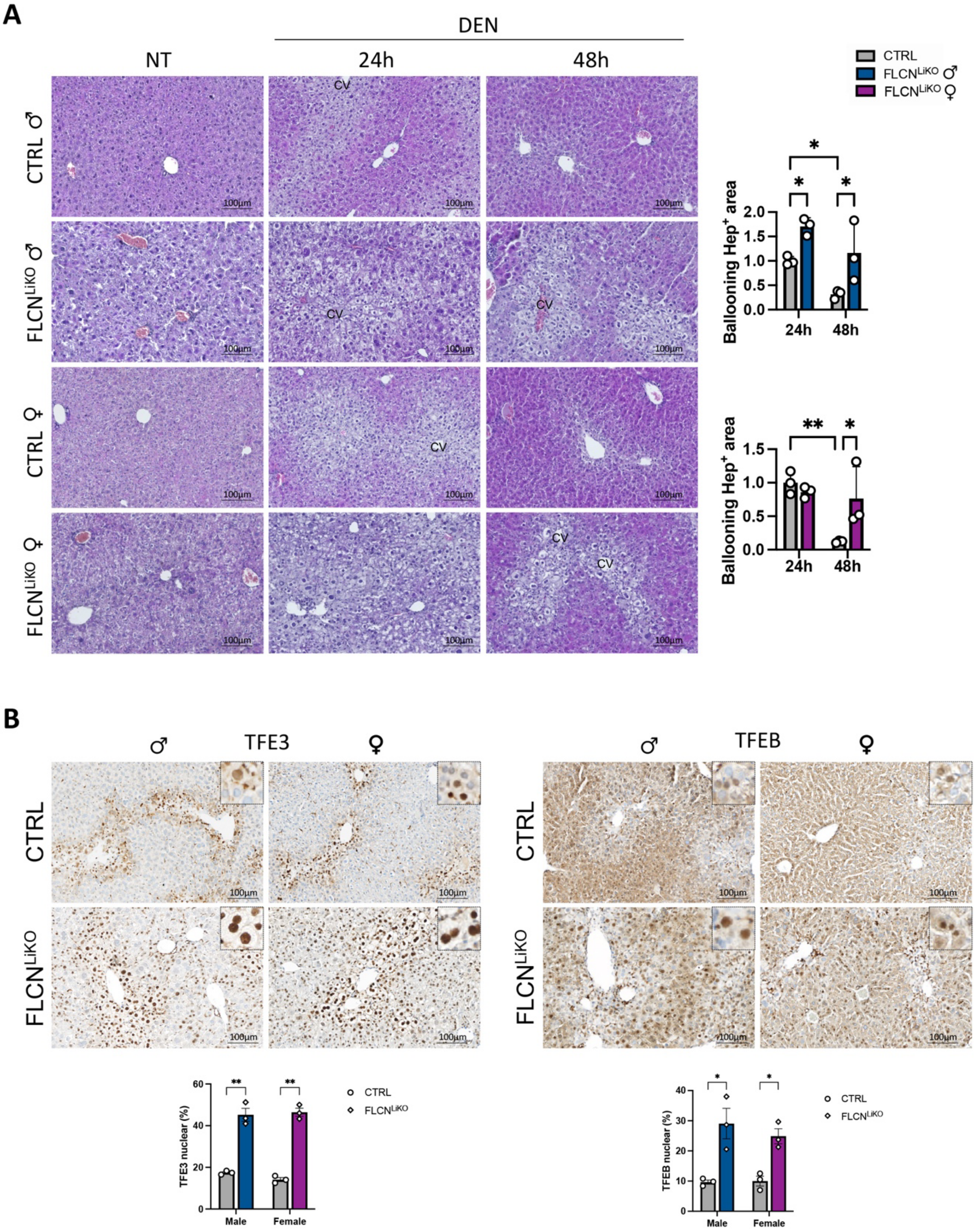
A
DEN
NT
24h
48h
CTRL ♂
FLCN^LiKO ♂
CTRL ♀
FLCN^LiKO ♀
CV
100µm
CTRL
FLCN^LiKO ♂
FLCN^LiKO ♀
Ballooning Hep+ area
24h
48h
B
TFE3
TFEB
♂
♀
CTRL
FLCN^LiKO
TFE3 nuclear (%)
TFEB nuclear (%)
Male
Female
CTRL
FLCN^LiKO

**Fig. S4. Flcn loss exacerbates cell damage following acute liver injury**

**A.** H&E staining of liver sections from $Flcn^{LiKO}$ and control male and female mice 24- and 48-hours post-injection of 100 mg/Kg of DEN, with relative quantification (n=3 per group). The data show an increased number of ballooning hepatocytes in $Flcn^{LiKO}$ mice. **B.** Immunostaining for TFE3 and TFEB in liver sections 48 hours post-injection, along with relative quantification (n=3 per group), indicating a similar response between male and female $Flcn^{LiKO}$ mice. CV, central vein. Each dot represents an individual mouse. Data are mean ± standard error. Statistical analysis: Two-way ANOVA: **p*-value < 0.05; ***p*-value < 0.01.

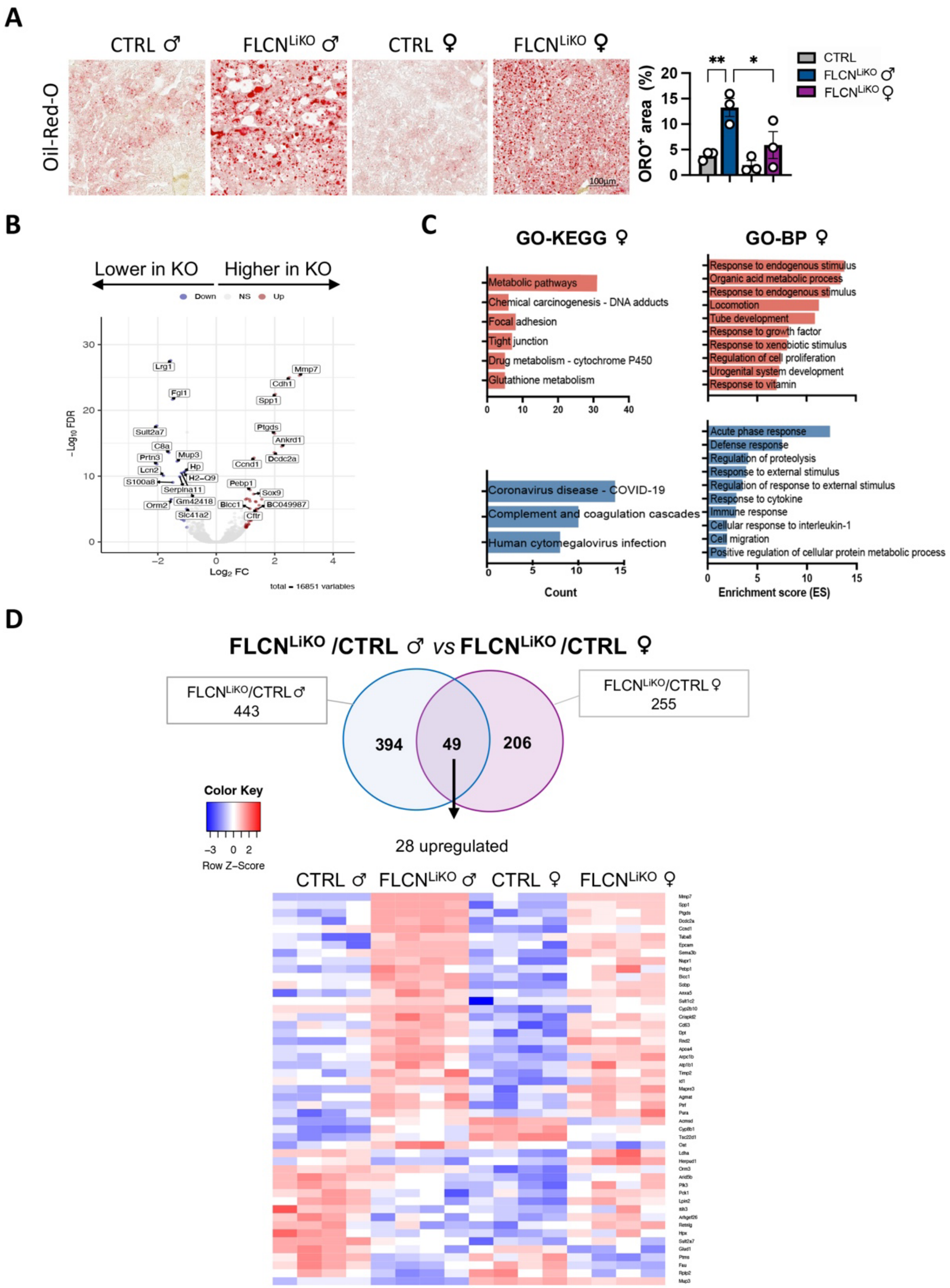

A
CTRL ♂
FLCN^LiKO ♂
CTRL ♀
FLCN^LiKO ♀
Oil-Red-O
100μm
ORO⁺ area (%)
CTRL
FLCN^LiKO ♂
FLCN^LiKO ♀
B
Lower in KO
Higher in KO
Down
NS
Up
−Log10 FDR
Log2 FC
total = 16851 variables
Lrg1
Fgl1
Sult2a7
C8a
Prtn3
Lcn2
S100a8
Mup3
Hp
H2-Q9
Serpina11
Orm2
Gm42418
Slc41a2
Mmp7
Cdh1
Spp1
Ptgds
Ankrd1
Dcdc2a
Ccnd1
Pebp1
Sox9
Bicc1
BC049987
Cftr
C
GO-KEGG ♀
GO-BP ♀
Metabolic pathways
Chemical carcinogenesis - DNA adducts
Focal adhesion
Tight junction
Drug metabolism - cytochrome P450
Glutathione metabolism
Coronavirus disease - COVID-19
Complement and coagulation cascades
Human cytomegalovirus infection
Count
Response to endogenous stimulus
Organic acid metabolic process
Response to endogenous stimulus
Locomotion
Tube development
Response to growth factor
Response to xenobiotic stimulus
Regulation of cell proliferation
Urogenital system development
Response to vitamin
Acute phase response
Defense response
Regulation of proteolysis
Response to external stimulus
Regulation of response to external stimulus
Response to cytokine
Immune response
Cellular response to interleukin-1
Cell migration
Positive regulation of cellular protein metabolic process
Enrichment score (ES)
D
FLCN^LiKO /CTRL ♂ vs FLCN^LiKO /CTRL ♀
FLCN^LiKO/CTRL ♂ 443
FLCN^LiKO/CTRL ♀ 255
394
49
206
28 upregulated
Color Key
Row Z-Score
CTRL ♂
FLCN^LiKO ♂
CTRL ♀
FLCN^LiKO ♀

**Fig. S5. Flcn$^{LiKO}$ female mice exhibit different transcriptomic changes compared to male mice.**

**A.** Oil-Red-O staining of liver sections from Flcn$^{LiKO}$ male and female mice, with relative quantification (n=3 per group), showing lipid accumulation following Flcn loss. **B.** Volcano plot of the differentially expressed genes (DEGs) between 90-week-old Flcn$^{LiKO}$ and control female mice. **C.** Gene Ontology (GO) analysis, including KEGG and Biological Process terms, highlighting significantly upregulated (red) and downregulated (blue) pathways in Flcn$^{LiKO}$ female mice compared to controls (FDR < 0.05). **D.** Venn diagram comparing transcriptomic datasets of Flcn$^{LiKO}$ male and female mice with their respective controls, along with a heatmap of the commonly upregulated or downregulated transcripts. Data are mean ± standard error. Statistical analysis: Two-way ANOVA: *$p$-value < 0.05; **$p$-value < 0.01.

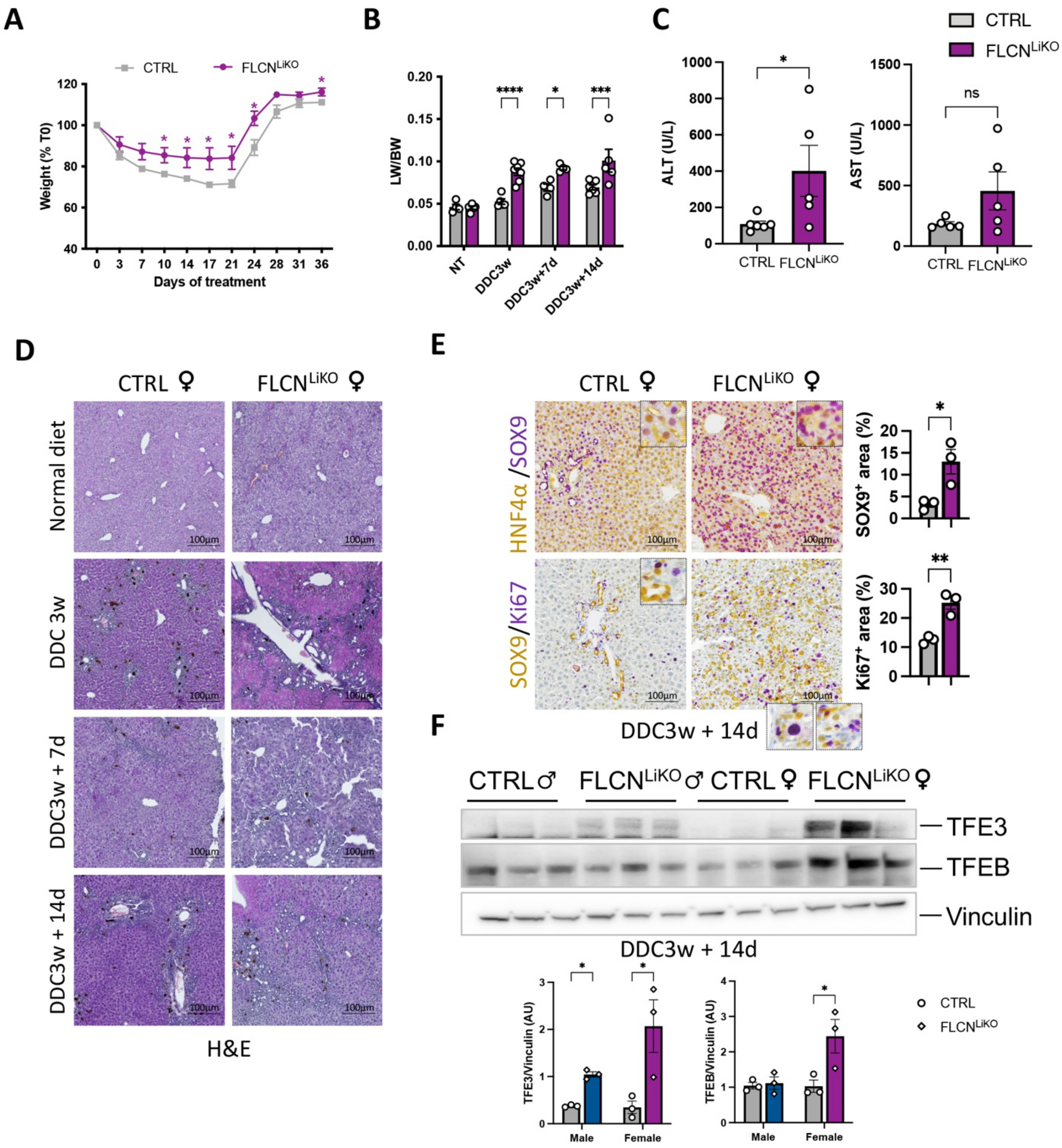
A
CTRL
FLCN^LiKO
Weight (% T0)
Days of treatment
B
LW/BW
NT
DDC3w
DDC3w+7d
DDC3w+14d
C
CTRL
FLCN^LiKO
ALT (U/L)
AST (U/L)
ns
D
CTRL ♀
FLCN^LiKO ♀
Normal diet
DDC 3w
DDC3w + 7d
DDC3w + 14d
H&E
E
HNF4α /SOX9
SOX9/Ki67
SOX9+ area (%)
Ki67+ area (%)
DDC3w + 14d
F
CTRL♂
FLCN^LiKO♂
CTRL♀
FLCN^LiKO♀
TFE3
TFEB
Vinculin
DDC3w + 14d
TFE3/Vinculin (AU)
TFEB/Vinculin (AU)
Male
Female

**Fig. S6. Comparable liver damage in Flcn$^{LiKO}$ male and female mice following DDC-induced injury and recovery**

**A.** Changes in body weight of Flcn$^{LiKO}$ and control female mice during the injury protocol (n=5 per group). **B.** Liver-to-body weight (LW/BW) ratio of mice of the indicated genotypes at the specified time points during liver injury and regeneration (n=5-7 per group). **C.** Serum ALT and AST levels of Flcn$^{LiKO}$ and control female mice two weeks after the removal of DDC-containing food (n=5 per group). **D, E.** Histological analysis of Flcn$^{LiKO}$ and control female mice at the indicated time points, with relative quantification (n=3 per group). **F.** Immunoblot analysis of TFE3 and TFEB in Flcn$^{LiKO}$ and control male and female mice 14 days after DDC removal, with relative quantification (n=3 per group). Each dot represents an individual mouse. Data are mean ± standard error. Statistical analysis: Two-way ANOVA or Student *t*-test: *$p$-value < 0.05.

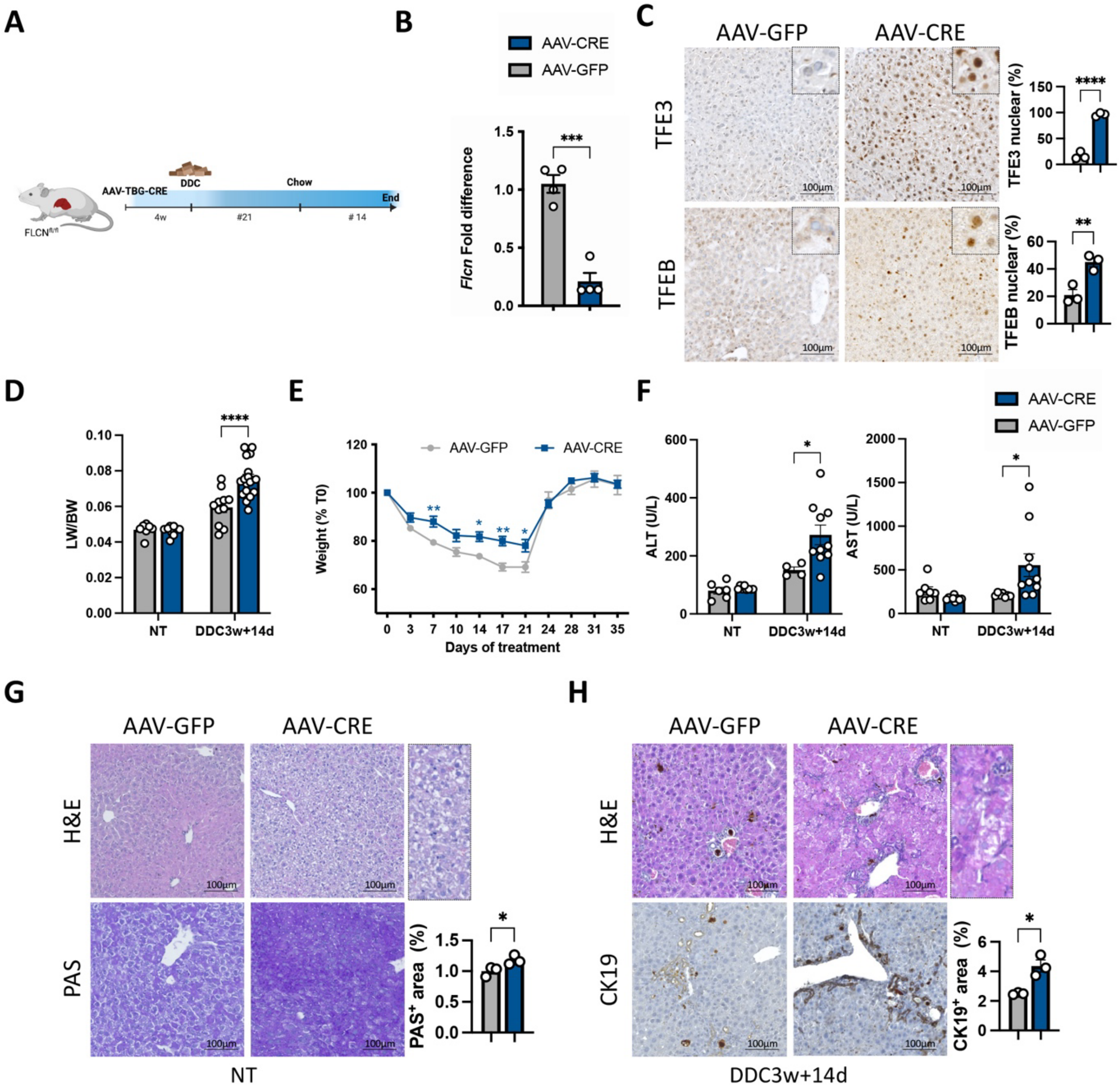

**Fig. S7. Hepatocyte-specific *Flcn* depletion leads to a milder liver phenotype**

**A.** Schematic representation of the experimental plan, illustrating AAV-TBG-CRE virus injection at 4 weeks of age, followed by a 4-week washout period before initiating treatment with a DDC-containing diet. **B.** *Flcn* expression levels after CRE recombination in $Flcn^{HepKO}$ and control mice (n=3 AAV-GFP and n=4 AAV-CRE). **C.** Immunostaining for TFEB and TFE3 in liver sections from mice 12 weeks post-injection with AAV-GFP or AAV-CRE viruses, with relative quantification (n=3 per group). **D.** Liver-to-body weight (LW/BW) ratio of mice of the indicated

genotypes before and after the liver injury protocol (n=7 AAV-GFP and n=9 AAV-CRE NT, n=10 AAV-GFP and n=20 AAV-CRE DDC3w+14d). **E.** Changes in body weight of $Flcn^{HepKO}$ and control mice during the injury protocol (n=5 AAV-GFP and n=10 AAV-CRE). **F.** Serum ALT and AST levels of $Flcn^{HepKO}$ and control mice before and two weeks after removal of the DDC-containing food (n=7 AAV-GFP and n=10 AAV-CRE). **G,H.** Histological analysis of $Flcn^{HepKO}$ and control mice before starting the DDC-containing diet (**G**) and at the end of the recovery phase (**H**), with relative quantification (n=3 per group). Each dot represents an individual mouse. Data are mean ± standard error. Statistical analysis: Two-way ANOVA or Student *t*-test: **p*-value < 0.05; ***p*-value < 0.01; *****p*-value < 0.0001.

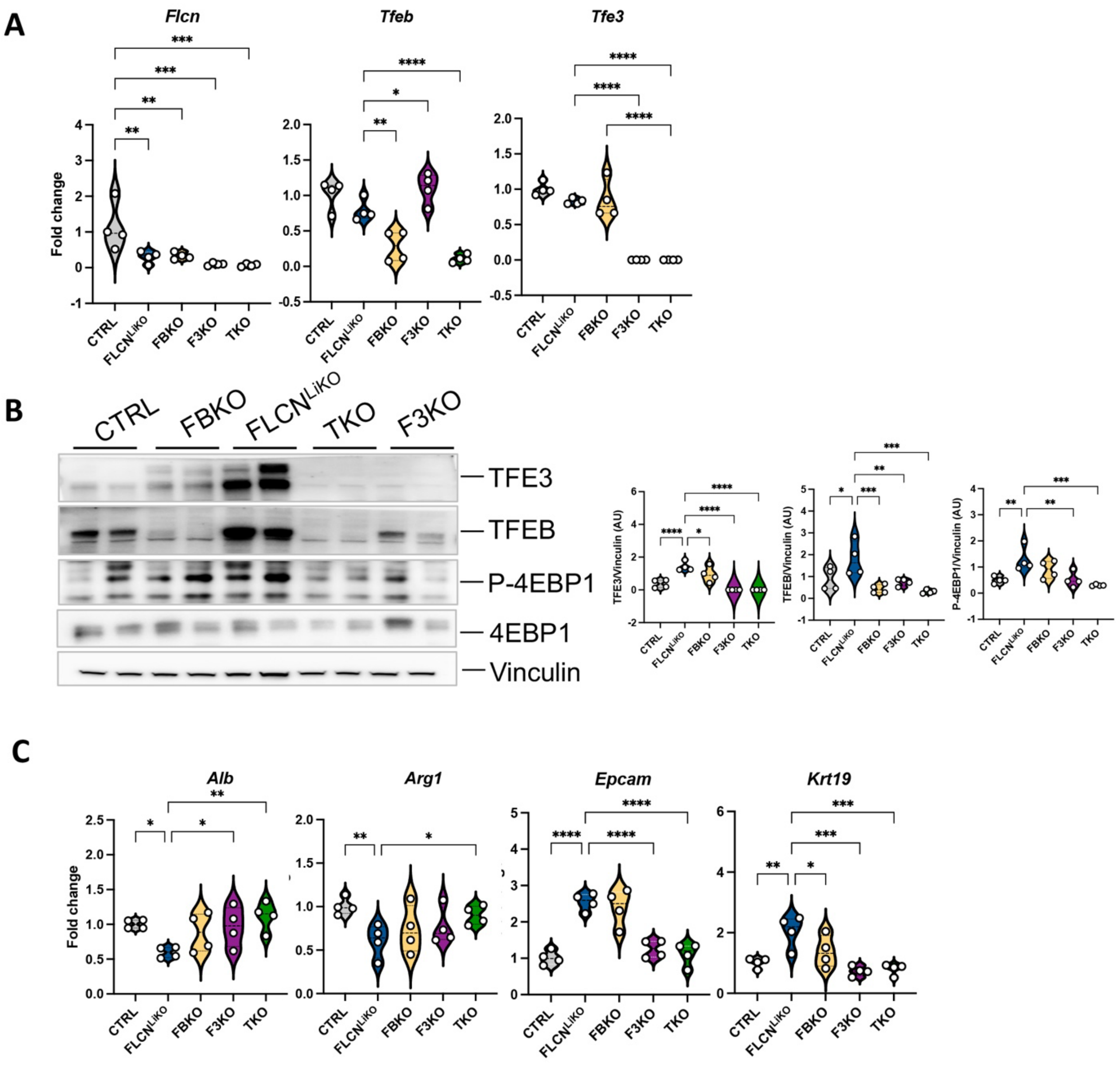


**Fig. S8. TFE3 depletion rescues liver abnormalities in Flcn$^{LiKO}$ mice (related to Figure 6).**

**A.** Gene expression analysis of *Flcn*, *Tfeb* and *Tfe3* in liver samples from control, Flcn$^{LiKO}$, FBKO, F3KO, and TKO mice 8 months after DEN injection (n=4 per group). **B.** Representative immunoblot analysis for TFEB, TFE3 and phosphorylated 4EBP1 (P-4EBP1) in total liver lysates from mice of the indicated genotypes 8 months after DEN injection, with relative quantification (n=4 per group). **C.** Gene expression analysis of liver samples from mice of the indicated genotypes (n=4 per group). All the data refer to male mice. Each dot represents an individual mouse. Data are mean ± standard error. Statistical analysis: One-way ANOVA: *$p$-value < 0.05; **$p$-value < 0.01; ***$p$-value < 0.001; ****$p$-value < 0.0001.

**Table S1.** GOEA and KEGG Pathway analyses performed on 646 and 480 DEGs in Flcn$^{LiKO}$ male mice vs CTRL 12-weeks old, restricting the output to KEGG pathways.

**INDUCED**

| Category | Term | Count | FDR |
|---|---|---|---|
| KEGG_PATHWAY | mmu04142:Lysosome | 41 | 2.04E-23 |
| KEGG_PATHWAY | mmu04145:Phagosome | 29 | 1.55E-08 |
| KEGG_PATHWAY | mmu01100:Metabolic pathways | 104 | 3.97E-07 |
| KEGG_PATHWAY | mmu05323:Rheumatoid arthritis | 17 | 6.71E-06 |
| KEGG_PATHWAY | mmu04966:Collecting duct acid secretion | 10 | 1.72E-05 |
| KEGG_PATHWAY | mmu00190:Oxidative phosphorylation | 19 | 1.05E-04 |
| KEGG_PATHWAY | mmu00600:Sphingolipid metabolism | 11 | 9.57E-04 |
| KEGG_PATHWAY | mmu05415:Diabetic cardiomyopathy | 22 | 0.001215457 |
| KEGG_PATHWAY | mmu04216:Ferroptosis | 9 | 0.002549465 |
| KEGG_PATHWAY | mmu00520:Amino sugar and nucleotide sugar metabolism | 10 | 0.002549465 |
| KEGG_PATHWAY | mmu04721:Synaptic vesicle cycle | 12 | 0.002924584 |
| KEGG_PATHWAY | mmu00511:Other glycan degradation | 6 | 0.009109127 |
| KEGG_PATHWAY | mmu00531:Glycosaminoglycan degradation | 6 | 0.018221381 |
| KEGG_PATHWAY | mmu05020:Prion disease | 22 | 0.019209023 |
| KEGG_PATHWAY | mmu00620:Pyruvate metabolism | 8 | 0.019783552 |
| KEGG_PATHWAY | mmu03320:PPAR signaling pathway | 11 | 0.028230914 |
| KEGG_PATHWAY | mmu05152:Tuberculosis | 16 | 0.045799735 |
| KEGG_PATHWAY | mmu04210:Apoptosis | 13 | 0.070617666 |
| KEGG_PATHWAY | mmu05166:Human T-cell leukemia virus 1 infection | 19 | 0.079072302 |
| KEGG_PATHWAY | mmu04612:Antigen processing and presentation | 10 | 0.082794485 |
| KEGG_PATHWAY | mmu04071:Sphingolipid signaling pathway | 12 | 0.082794485 |

**INHIBITED**

| Category | Term | Count | FDR |
|---|---|---|---|
| KEGG_PATHWAY | mmu01100:Metabolic pathways | 91 | 8.57E-12 |
| KEGG_PATHWAY | mmu00140:Steroid hormone biosynthesis | 20 | 2.06E-10 |
| KEGG_PATHWAY | mmu05204:Chemical carcinogenesis - DNA adducts | 16 | 2.40E-07 |
| KEGG_PATHWAY | mmu00983:Drug metabolism - other enzymes | 15 | 5.14E-06 |
| KEGG_PATHWAY | mmu04610:Complement and coagulation cascades | 14 | 3.78E-05 |
| KEGG_PATHWAY | mmu00982:Drug metabolism - cytochrome P450 | 11 | 5.22E-04 |
| KEGG_PATHWAY | mmu00591:Linoleic acid metabolism | 9 | 0.001173698 |
| KEGG_PATHWAY | mmu00830:Retinol metabolism | 12 | 0.001173698 |
| KEGG_PATHWAY | mmu04976:Bile secretion | 12 | 0.001388144 |
| KEGG_PATHWAY | mmu00980:Metabolism of xenobiotics by cytochrome P450 | 10 | 0.002511303 |
| KEGG_PATHWAY | mmu04726:Serotonergic synapse | 13 | 0.003139527 |
| KEGG_PATHWAY | mmu04979:Cholesterol metabolism | 8 | 0.005659425 |
| KEGG_PATHWAY | mmu00590:Arachidonic acid metabolism | 9 | 0.027418402 |
| KEGG_PATHWAY | mmu04750:Inflammatory mediator regulation of TRP channels | 11 | 0.027418402 |
| KEGG_PATHWAY | mmu04146:Peroxisome | 9 | 0.027418402 |
| KEGG_PATHWAY | mmu00770:Pantothenate and CoA biosynthesis | 5 | 0.027712917 |
| KEGG_PATHWAY | mmu01240:Biosynthesis of cofactors | 12 | 0.027712917 |
| KEGG_PATHWAY | mmu05207:Chemical carcinogenesis - receptor activation | 15 | 0.027712917 |
| KEGG_PATHWAY | mmu00380:Tryptophan metabolism | 7 | 0.027712917 |
| KEGG_PATHWAY | mmu00260:Glycine, serine and threonine metabolism | 6 | 0.043584925 |
| KEGG_PATHWAY | mmu05418:Fluid shear stress and atherosclerosis | 11 | 0.055996562 |

**Table S2.** GOEA and KEGG Pathway analyses performed on 136 and 191 DEGs in Flcn$^{LiKO}$ female mice vs CTR at 12-weeks of age, restricting the output to KEGG pathways**.**

## INDUCED

| Category | Term | Count | FDR |
|---|---|---|---|
| KEGG_PATHWAY | mmu05210:Colorectal cancer | 6 | 0.038338333 |
| KEGG_PATHWAY | mmu03320:PPAR signaling pathway | 6 | 0.038338333 |

## INHIBITED

| Category | Term | Count | FDR |
|---|---|---|---|
| KEGG_PATHWAY | mmu03010:Ribosome | 21 | 1.61E-11 |
| KEGG_PATHWAY | mmu05171:Coronavirus disease - COVID-19 | 23 | 6.88E-11 |
| KEGG_PATHWAY | mmu01100:Metabolic pathways | 51 | 2.56E-08 |
| KEGG_PATHWAY | mmu04610:Complement and coagulation cascades | 12 | 1.70E-06 |
| KEGG_PATHWAY | mmu04146:Peroxisome | 8 | 0.005291745 |
| KEGG_PATHWAY | mmu00983:Drug metabolism - other enzymes | 7 | 0.044635281 |

**Table S3.** Comparison of 12- weeks- old FLCN$^{LiKO}$ mice vs control both in males and females. 152 DEGs are commonly regulated, 974 remained specifically regulated in males and 175 in females.

| Symbol | log2FoldChange_YFKO_YF | padj_YFKO_YF | log2FoldChange_YMKO_YM | padj_YMKO_YM |
|---|---|---|---|---|
| Hnf4aos | -1.0919 | 0.0184 | -2.3191 | 0.0000 |
| Rgs16 | -1.1968 | 0.0047 | -1.7578 | 0.0000 |
| Ces3b | -1.1468 | 0.0002 | -1.4442 | 0.0000 |
| Mup3 | -1.0679 | 0.0000 | -1.3686 | 0.0000 |
| 1600002H07Rik | -1.0525 | 0.0079 | -1.1305 | 0.0023 |
| Nxpe2 | -0.8746 | 0.0061 | -1.0544 | 0.0000 |
| Egfr | -0.5880 | 0.0217 | -0.9684 | 0.0225 |
| Cyp27a1 | -0.5335 | 0.0129 | -0.9297 | 0.0000 |
| Ces2a | -0.7351 | 0.0058 | -0.8818 | 0.0000 |
| Cyp2c44 | -0.4755 | 0.0231 | -0.8654 | 0.0000 |
| Dcxr | -0.7123 | 0.0072 | -0.8650 | 0.0001 |
| Serpina1c | -1.2834 | 0.0000 | -0.8257 | 0.0000 |
| Keg1 | -0.9194 | 0.0008 | -0.7749 | 0.0004 |
| Stard10 | -0.8276 | 0.0021 | -0.7441 | 0.0000 |
| Ndrg2 | -0.7578 | 0.0020 | -0.7425 | 0.0001 |
| Stard4 | -0.5696 | 0.0256 | -0.7273 | 0.0001 |
| Glud1 | -0.5786 | 0.0184 | -0.7064 | 0.0000 |
| Rpl18a | -0.9279 | 0.0032 | -0.6983 | 0.0003 |
| Ces1e | -0.5060 | 0.0196 | -0.6903 | 0.0000 |
| Mvk | -0.8576 | 0.0046 | -0.6807 | 0.0054 |
| Sdc4 | -0.5569 | 0.0169 | -0.6726 | 0.0012 |
| Hes6 | -0.6826 | 0.0455 | -0.6619 | 0.0066 |
| Rcl1 | -0.8333 | 0.0054 | -0.6589 | 0.0001 |
| Tk1 | -0.6985 | 0.0449 | -0.6555 | 0.0234 |
| Malat1 | 0.8376 | 0.0298 | -0.6540 | 0.0002 |
| Paics | -0.7026 | 0.0070 | -0.6291 | 0.0008 |
| Serpina1a | -0.6829 | 0.0231 | -0.6274 | 0.0008 |
| Serpina1d | -0.6604 | 0.0156 | -0.6138 | 0.0000 |
| Ceacam1 | -0.4676 | 0.0473 | -0.6124 | 0.0002 |
| Khk | -0.8244 | 0.0000 | -0.6114 | 0.0002 |
| Pltp | -0.6975 | 0.0156 | -0.6093 | 0.0445 |
| Map3k5 | -0.6686 | 0.0375 | -0.6051 | 0.0312 |
| Nr1d1 | -1.0099 | 0.0000 | -0.6028 | 0.0027 |
| Ambp | -0.8232 | 0.0002 | -0.5923 | 0.0002 |
| Dpys | -0.5842 | 0.0308 | -0.5922 | 0.0006 |
| Haao | -0.5775 | 0.0047 | -0.5643 | 0.0006 |
| Gstm1 | -0.7644 | 0.0001 | -0.5553 | 0.0061 |
| Cyp4f14 | -0.6415 | 0.0072 | -0.5531 | 0.0134 |
| Ece1 | -0.5911 | 0.0057 | -0.5497 | 0.0011 |
| Pecr | -0.8154 | 0.0044 | -0.5432 | 0.0047 |
| Gjb1 | -0.6321 | 0.0006 | -0.5345 | 0.0001 |
| Sardh | -0.4623 | 0.0204 | -0.5283 | 0.0015 |
| Pon1 | -0.6292 | 0.0165 | -0.5228 | 0.0085 |
| Tecr | -0.7612 | 0.0160 | -0.5198 | 0.0326 |
| Trmt112 | -0.5985 | 0.0284 | -0.5128 | 0.0085 |
| Paox | -0.7566 | 0.0047 | -0.5111 | 0.0298 |

| | | | | |
|---|---|---|---|---|
| Bbox1 | -0.5803 | 0.0097 | -0.5081 | 0.0214 |
| Chp1 | -0.5981 | 0.0276 | -0.4994 | 0.0030 |
| Lrp1 | -0.6449 | 0.0058 | -0.4977 | 0.0010 |
| Vtn | -0.4784 | 0.0165 | -0.4934 | 0.0009 |
| Uox | -0.5504 | 0.0167 | -0.4849 | 0.0038 |
| Asgr1 | -0.6583 | 0.0035 | -0.4781 | 0.0001 |
| Tkfc | -0.7344 | 0.0011 | -0.4765 | 0.0296 |
| Serpina1b | -0.8136 | 0.0156 | -0.4728 | 0.0076 |
| Msmo1 | -0.6253 | 0.0365 | -0.4699 | 0.0136 |
| B630019A10Rik | -0.6601 | 0.0023 | -0.4584 | 0.0362 |
| Slc2a9 | -0.6197 | 0.0194 | -0.4566 | 0.0460 |
| Gltscr2 | -0.6737 | 0.0112 | -0.4543 | 0.0130 |
| Aup1 | -0.5831 | 0.0231 | -0.4518 | 0.0174 |
| Papss2 | -0.5249 | 0.0401 | -0.4460 | 0.0358 |
| F12 | -0.6528 | 0.0184 | -0.4381 | 0.0309 |
| Slc25a1 | -0.4875 | 0.0400 | -0.4236 | 0.0422 |
| Hal | -0.6226 | 0.0156 | -0.4131 | 0.0211 |
| Rps26 | -0.7484 | 0.0058 | -0.4035 | 0.0215 |
| Fau | -0.6006 | 0.0097 | -0.3917 | 0.0149 |
| Tprkb | -0.7792 | 0.0000 | -0.3826 | 0.0413 |
| Plg | -0.5979 | 0.0375 | -0.3127 | 0.0149 |
| Cyp3a11 | 1.1261 | 0.0000 | 0.4888 | 0.0061 |
| Rpl41 | -0.6505 | 0.0461 | 0.5369 | 0.0086 |
| Zfyve26 | 0.7125 | 0.0195 | 0.6731 | 0.0063 |
| Cldn1 | 0.6749 | 0.0071 | 0.6847 | 0.0055 |
| Trim2 | 0.7461 | 0.0194 | 0.6931 | 0.0022 |
| Gas2 | 0.9462 | 0.0022 | 0.7485 | 0.0042 |
| Appl1 | 0.6776 | 0.0301 | 0.7813 | 0.0022 |
| Ywhah | 0.6095 | 0.0156 | 0.7874 | 0.0000 |
| Bax | 1.1002 | 0.0048 | 0.7877 | 0.0170 |
| Arsa | 0.8825 | 0.0291 | 0.8059 | 0.0016 |
| Cybb | 0.6696 | 0.0347 | 0.8894 | 0.0003 |
| Rnf144a | 0.7507 | 0.0301 | 0.9015 | 0.0124 |
| Snhg11 | 2.2043 | 0.0000 | 0.9247 | 0.0112 |
| Chka | 1.0310 | 0.0046 | 0.9489 | 0.0002 |
| P2rx4 | 0.8161 | 0.0135 | 0.9584 | 0.0001 |
| H2-Ab1 | 0.8594 | 0.0070 | 0.9677 | 0.0012 |
| Ankrd12 | 0.9742 | 0.0028 | 0.9773 | 0.0001 |
| Pfkp | 0.9749 | 0.0469 | 1.0120 | 0.0146 |
| Cyp4a12b | 2.5474 | 0.0000 | 1.0211 | 0.0000 |
| Plekha1 | 0.9651 | 0.0088 | 1.0608 | 0.0002 |
| Adora1 | 0.8880 | 0.0209 | 1.0793 | 0.0001 |
| Tmem116 | 1.0924 | 0.0156 | 1.1189 | 0.0155 |
| Lipa | 0.7686 | 0.0008 | 1.2048 | 0.0000 |
| Ifi27l2b | 1.1116 | 0.0055 | 1.2167 | 0.0125 |
| Ccnd1 | 0.5545 | 0.0285 | 1.2366 | 0.0000 |
| Tmem86a | 1.1716 | 0.0009 | 1.2435 | 0.0001 |
| Saa1 | 1.3391 | 0.0000 | 1.2500 | 0.0000 |
| Cpne8 | 1.3349 | 0.0023 | 1.2765 | 0.0017 |
| Appl2 | 0.9236 | 0.0027 | 1.2893 | 0.0000 |
| Slc16a7 | 0.7819 | 0.0002 | 1.3326 | 0.0000 |
| Spp1 | 1.4659 | 0.0000 | 1.3428 | 0.0000 |
| Ccdc34 | 1.2158 | 0.0013 | 1.3507 | 0.0000 |

| | | | | |
|---|---|---|---|---|
| Acot2 | 0.7784 | 0.0412 | 1.3669 | 0.0000 |
| Lpl | 1.1236 | 0.0019 | 1.3752 | 0.0000 |
| Ctsa | 0.7820 | 0.0002 | 1.4208 | 0.0000 |
| Ctsz | 0.6158 | 0.0096 | 1.4328 | 0.0000 |
| Gpx3 | 1.0324 | 0.0448 | 1.4688 | 0.0000 |
| Ttc3 | 0.7076 | 0.0076 | 1.4765 | 0.0000 |
| Gldn | 2.0848 | 0.0000 | 1.5068 | 0.0014 |
| Plin3 | 0.7571 | 0.0173 | 1.6470 | 0.0000 |
| Saa2 | 1.1882 | 0.0001 | 1.6786 | 0.0000 |
| Hexa | 1.1182 | 0.0000 | 1.6968 | 0.0000 |
| Gdf15 | 0.9127 | 0.0000 | 1.7308 | 0.0000 |
| Anxa5 | 0.7553 | 0.0245 | 1.7425 | 0.0000 |
| Cfap44 | 0.9472 | 0.0347 | 1.8017 | 0.0000 |
| Plekhg5 | 1.1297 | 0.0013 | 1.8451 | 0.0000 |
| Pdk4 | 1.1912 | 0.0112 | 1.8856 | 0.0000 |
| Clec2h | 1.2812 | 0.0030 | 1.9177 | 0.0000 |
| Lcn2 | 1.1269 | 0.0044 | 2.0434 | 0.0000 |
| Dpp7 | 1.0744 | 0.0021 | 2.0494 | 0.0000 |
| D7Ertd443e | 1.2726 | 0.0024 | 2.0822 | 0.0000 |
| Gm31522 | 1.1495 | 0.0160 | 2.0887 | 0.0000 |
| Evc | 1.1274 | 0.0043 | 2.1368 | 0.0000 |
| Gnai1 | 0.9645 | 0.0310 | 2.1575 | 0.0000 |
| Tifa | 1.1325 | 0.0039 | 2.1600 | 0.0000 |
| Kcnk3 | 3.0073 | 0.0000 | 2.2229 | 0.0000 |
| Clstn3 | 1.7426 | 0.0000 | 2.2850 | 0.0000 |
| Cd36 | 2.0841 | 0.0000 | 2.3724 | 0.0000 |
| Sema3b | 1.0544 | 0.0347 | 2.4789 | 0.0000 |
| 2010003K11Rik | 1.2220 | 0.0002 | 2.5260 | 0.0000 |
| Ly6d | 2.6218 | 0.0000 | 2.6901 | 0.0000 |
| Blnk | 1.4056 | 0.0007 | 2.6977 | 0.0000 |
| Slpi | 1.4215 | 0.0006 | 2.7049 | 0.0000 |
| Chil1 | 1.0127 | 0.0315 | 2.9235 | 0.0000 |
| Cib3 | 1.4574 | 0.0006 | 2.9569 | 0.0000 |
| Gm10804 | 1.4280 | 0.0009 | 2.9823 | 0.0000 |
| Osbpl3 | 1.3206 | 0.0020 | 3.0517 | 0.0000 |
| AI314278 | 1.2564 | 0.0026 | 3.0672 | 0.0000 |
| Slc35f2 | 0.9082 | 0.0461 | 3.0887 | 0.0000 |
| Gpnmb | 2.1575 | 0.0000 | 3.1880 | 0.0000 |
| Tuba8 | 1.9898 | 0.0000 | 3.2601 | 0.0000 |
| Nupr1 | 1.5507 | 0.0000 | 3.3957 | 0.0000 |
| Renbp | 1.4894 | 0.0000 | 3.4287 | 0.0000 |
| Bex2 | 2.5695 | 0.0000 | 3.5432 | 0.0000 |
| Cln6 | 2.1855 | 0.0000 | 3.5676 | 0.0000 |
| Cyp46a1 | 2.6960 | 0.0000 | 3.5773 | 0.0000 |
| Uap1l1 | 1.5869 | 0.0000 | 3.6424 | 0.0000 |
| Wfdc2 | 2.0108 | 0.0000 | 3.8291 | 0.0000 |
| Apoa4 | 1.0414 | 0.0411 | 3.9344 | 0.0000 |
| Rragd | 1.4198 | 0.0009 | 4.0093 | 0.0000 |
| Ephb2 | 1.7498 | 0.0000 | 4.0369 | 0.0000 |
| Fabp3 | 2.5008 | 0.0000 | 4.2379 | 0.0000 |
| Cd63 | 2.2331 | 0.0000 | 4.2779 | 0.0000 |
| Plin4 | 1.7014 | 0.0000 | 4.3471 | 0.0000 |
| Mmp27 | 3.1841 | 0.0000 | 4.9023 | 0.0000 |

**Table S4.** GOEA and KEGG pathway analysis on transcripts commonly and specifically regulated in 12- weeks- old $FLCN^{LiKO}$ mice vs control males and females (refer to Supplementary Table 3).

**INDUCED**

| **Category** | **Term** | **Count** | **FDR** |
|---|---|---|---|
| KEGG_PATHWAY | mmu04142:Lysosome | 6 | 0.077282598 |
| KEGG_PATHWAY | mmu03320:PPAR signaling pathway | 5 | 0.077282598 |

**INHIBITED**

| **Category** | **Term** | **Count** | **FDR** |
|---|---|---|---|
| KEGG_PATHWAY | mmu04610:Complement and coagulation cascades | 7 | 4.15E-04 |
| KEGG_PATHWAY | mmu01100:Metabolic pathways | 19 | 0.006006153 |
| KEGG_PATHWAY | mmu00983:Drug metabolism - other enzymes | 5 | 0.02907789 |

**Table S5.** GOEA and KEGG Pathway analyses performed on 259 and 184 DEGs in Flcn$^{LiKO}$ male mice vs CTR at 90-weeks of age, restricting the output to KEGG pathways.

## INDUCED

| Category | Term | Count | FDR |
|---|---|---|---|
| KEGG_PATHWAY | mmu04066:HIF-1 signaling pathway | 9 | 0.049375723 |
| KEGG_PATHWAY | mmu03320:PPAR signaling pathway | 8 | 0.049375723 |
| KEGG_PATHWAY | mmu04152:AMPK signaling pathway | 9 | 0.057604758 |

## INHIBITED

| Category | Term | Count | FDR |
|---|---|---|---|
| KEGG_PATHWAY | mmu01100:Metabolic pathways | 43 | 1.50E-05 |
| KEGG_PATHWAY | mmu00140:Steroid hormone biosynthesis | 9 | 0.001261256 |
| KEGG_PATHWAY | mmu00910:Nitrogen metabolism | 5 | 0.002745675 |
| KEGG_PATHWAY | mmu05204:Chemical carcinogenesis - DNA adducts | 7 | 0.023273542 |

**Table S6.** GOEA and KEGG Pathway analyses performed on 143 and 112 DEGs in Flcn$^{LiKO}$ female mice vs CTR at 90-weeks of age, restricting the output to KEGG pathways.

## INDUCED

| Category | Term | Count | FDR |
|---|---|---|---|
| KEGG_PATHWAY | mmu01100:Metabolic pathways | 31 | 0.001498559 |
| KEGG_PATHWAY | mmu05204:Chemical carcinogenesis - DNA adducts | 6 | 0.061451022 |
| KEGG_PATHWAY | mmu04510:Focal adhesion | 8 | 0.087853641 |
| KEGG_PATHWAY | mmu04530:Tight junction | 7 | 0.092977668 |
| KEGG_PATHWAY | mmu00982:Drug metabolism - cytochrome P450 | 5 | 0.092977668 |
| KEGG_PATHWAY | mmu00480:Glutathione metabolism | 5 | 0.092977668 |

## INHIBITED

| Category | Term | Count | FDR |
|---|---|---|---|
| KEGG_PATHWAY | mmu05171:Coronavirus disease - COVID-19 | 14 | 9.30E-07 |
| KEGG_PATHWAY | mmu04610:Complement and coagulation cascades | 10 | 9.30E-07 |
| KEGG_PATHWAY | mmu05163:Human cytomegalovirus infection | 8 | 0.091304388 |

**Table S7.** Comparison of 90- weeks- old $FLCN^{LiKO}$ mice vs control both in males and females. 49 DEGs are commonly regulated, 394 remained specifically regulated in males and 206 in females.

| Symbol | log2FoldChange_O_F_KO vs O_F | padj_O_F_KO vs O_F | log2FoldChange_O_M_KO vs O_M | padj_O_M_KO vs O_M |
|---|---|---|---|---|
| Tsc22d1 | 0.615 | 0.013 | -1.184 | 0.000 |
| Acmsd | 1.036 | 0.000 | -0.889 | 0.023 |
| Mup3 | -1.339 | 0.000 | -0.810 | 0.000 |
| Rplp2 | -0.547 | 0.008 | -0.552 | 0.020 |
| Cyp8b1 | 0.649 | 0.006 | -0.521 | 0.000 |
| Fau | -0.534 | 0.003 | -0.505 | 0.005 |
| Ptms | -0.467 | 0.003 | -0.460 | 0.026 |
| Oat | 0.595 | 0.005 | -0.441 | 0.025 |
| Glud1 | -0.426 | 0.013 | -0.393 | 0.045 |
| Pura | 0.428 | 0.031 | 0.475 | 0.010 |
| Agmat | 0.497 | 0.040 | 0.510 | 0.007 |
| Pck1 | -0.706 | 0.002 | 0.525 | 0.041 |
| Timp2 | 0.603 | 0.028 | 0.577 | 0.027 |
| Mapre3 | 0.526 | 0.023 | 0.626 | 0.020 |
| Arpc1b | 0.622 | 0.000 | 0.632 | 0.012 |
| Anxa5 | 1.048 | 0.000 | 0.642 | 0.002 |
| Hpx | -0.883 | 0.000 | 0.652 | 0.012 |
| Ptrf | 0.491 | 0.044 | 0.653 | 0.007 |
| Itih3 | -0.829 | 0.025 | 0.682 | 0.034 |
| Ldha | -0.461 | 0.000 | 0.721 | 0.001 |
| Pebp1 | 1.156 | 0.000 | 0.754 | 0.020 |
| Arid5b | -0.645 | 0.004 | 0.755 | 0.005 |
| Arhgef26 | -0.832 | 0.007 | 0.757 | 0.012 |
| Atp1b1 | 0.614 | 0.005 | 0.758 | 0.000 |
| Rnd2 | 0.779 | 0.000 | 0.770 | 0.000 |
| Sobp | 1.101 | 0.002 | 0.802 | 0.049 |
| Lpin2 | -0.745 | 0.001 | 0.910 | 0.000 |
| Sult2a7 | -2.040 | 0.000 | 0.966 | 0.039 |
| Plk3 | -0.693 | 0.019 | 0.971 | 0.006 |
| Sema3b | 1.232 | 0.000 | 0.976 | 0.017 |
| Herpud1 | -0.605 | 0.005 | 0.977 | 0.000 |
| Nupr1 | 1.165 | 0.000 | 0.993 | 0.030 |
| Crispld2 | 0.947 | 0.013 | 1.020 | 0.019 |
| Epcam | 1.237 | 0.000 | 1.051 | 0.016 |
| Bicc1 | 1.150 | 0.000 | 1.061 | 0.007 |
| Sult1c2 | 1.026 | 0.002 | 1.081 | 0.013 |
| Dpt | 0.828 | 0.023 | 1.083 | 0.009 |
| Tuba8 | 1.252 | 0.000 | 1.092 | 0.013 |
| Cd63 | 0.870 | 0.001 | 1.098 | 0.000 |
| Cyp2b10 | 0.955 | 0.000 | 1.161 | 0.005 |
| Retnlg | -0.877 | 0.042 | 1.161 | 0.004 |
| Apoa4 | 0.778 | 0.000 | 1.163 | 0.000 |
| Orm3 | -0.625 | 0.016 | 1.210 | 0.004 |
| Dcdc2a | 1.987 | 0.000 | 1.338 | 0.000 |
| Ccnd1 | 1.291 | 0.000 | 1.432 | 0.000 |
| Ptgds | 1.994 | 0.000 | 1.452 | 0.000 |

| | | | | |
|---|---|---|---|---|
| Id1 | 0.571 | 0.040 | 1.554 | 0.000 |
| Spp1 | 2.020 | 0.000 | 1.612 | 0.000 |
| Mmp7 | 2.848 | 0.000 | 1.666 | 0.000 |

**Table S8.** GOEA and KEGG pathway analysis on transcripts specifically regulated in 90- weeks-old FLCN$^{LiKO}$ mice vs control males and females.

## SPECIFICALLY INDUCED IN FEMALES

| Category | Term | Count | FDR |
|---|---|---|---|
| KEGG_PATHWAY | mmu01100:Metabolic pathways | 25 | 0.005749838 |
| KEGG_PATHWAY | mmu05204:Chemical carcinogenesis - DNA adducts | 6 | 0.016424063 |
| KEGG_PATHWAY | mmu00982:Drug metabolism - cytochrome P450 | 5 | 0.047740618 |
| KEGG_PATHWAY | mmu00480:Glutathione metabolism | 5 | 0.047740618 |

## SPECIFICALLY INHIBITED IN FEMALES

| Category | Term | Count | FDR |
|---|---|---|---|
| KEGG_PATHWAY | mmu04610:Complement and coagulation cascades | 10 | 3.18E-07 |
| KEGG_PATHWAY | mmu05171:Coronavirus disease - COVID-19 | 12 | 7.47E-06 |
| KEGG_PATHWAY | mmu05163:Human cytomegalovirus infection | 8 | 0.027544218 |
| KEGG_PATHWAY | mmu05322:Systemic lupus erythematosus | 6 | 0.055394406 |
| KEGG_PATHWAY | mmu04657:IL-17 signaling pathway | 5 | 0.055394406 |

## SPECIFICALLY INHIBITED IN MALES

| Category | Term | Count | FDR |
|---|---|---|---|
| KEGG_PATHWAY | mmu01100:Metabolic pathways | 39 | 2.13E-04 |
| KEGG_PATHWAY | mmu00140:Steroid hormone biosynthesis | 9 | 8.07E-04 |
| KEGG_PATHWAY | mmu05204:Chemical carcinogenesis - DNA adducts | 7 | 0.022407134 |
| KEGG_PATHWAY | mmu00910:Nitrogen metabolism | 4 | 0.043854467 |

**Table S9.** *Primers used for real time PCR.*

| **Gene** | **Species** | | **Sequence (5'→ 3')** |
|---|---|---|---|
| *Ribosomal protein S16* | mouse | forward | AGGAGCGATTTGCTGGTGTGG |
| | | reverse | GCTACCAGGGCCTTTGAGATG |
| *Flcn* | mouse | forward | GGCTGTCAGAGCTCTGTTCCC |
| | | reverse | CTCTGACCCACTGCAGCCAC |
| *Tfe3* | mouse | forward | AGGATCAAAGAGCTGGGCAC |
| | | reverse | CCGGCTCTCCAGGTCTTTG |
| *Tfeb* | mouse | forward | CTGAACGTGTACAGCGGTGA |
| | | reverse | GTGATTGTCTTTCTTCTGCCGC |
| *Sox9* | mouse | forward | CGAGGAAGATAAGTTCCCCGTGTGC |
| | | reverse | CAGCAGCCTCCAGAGCTTGCCC |
| *Hnf4α* | mouse | forward | TGCCTGCCTCAAAGCCAT |
| | | reverse | CACTCAGCCCCTTGGCAT |
| *Gpnmb* | mouse | forward | TGCCAAGCGATTTCGTGATGT |
| | | reverse | GCCACGTAATTGGTTGTGCTC |
| *Rragd* | mouse | forward | GAGGTCACAAAGTTCTTGGCGC |
| | | reverse | CCTCGAAGACTTCATGGATGG |
| *Epcam* | mouse | forward | AACACAAGACGACGTGGACA |
| | | reverse | GCTCTCCGTTCACTCTCAGG |
| *Krt19* | mouse | forward | TGCTGGATGAGCTGACTCTG |
| | | reverse | AATCCACCTCCACACTGACC |
| *Alb* | mouse | forward | GCTGAGGCCATGTGCACCTCC |
| | | reverse | CCATCAAGCTTCGGGGTCAGG |
| *Arg1* | mouse | forward | CTCCAAGCCAAAGTCCTTAGAG |
| | | reverse | AGGAGCTGTCATTAGGGACATC |

**Table S10.** *Primary antibodies used for immunoblots and stainings.*

| Antigen | Species in which the Ab was raised | Source | Code |
|---|---|---|---|
| TFEB | Rabbit | Bethyl | A303-673A |
| TFE3 | Rabbit | Sigma | HPA023881 |
| SOX9 | Rabbit | Millipore | Ab5535 |
| HNF4α | Mouse | Abcam | Ab41898 |
| CK19 | Rabbit | Abcam | AB133496 |
| P-S6 | Rabbit | Cell Signaling | CST-5364 |
| F4/80 | Rabbit | Abcam | Ab6640 |
| Ki67 | Rabbit | Abcam | ab16667 |
| P-4EBP1 | Rabbit | Cell Signaling | CST-9451 |
| 4EBP1 | Rabbit | Cell Signaling | CST-9644 |

## Original data

**A**

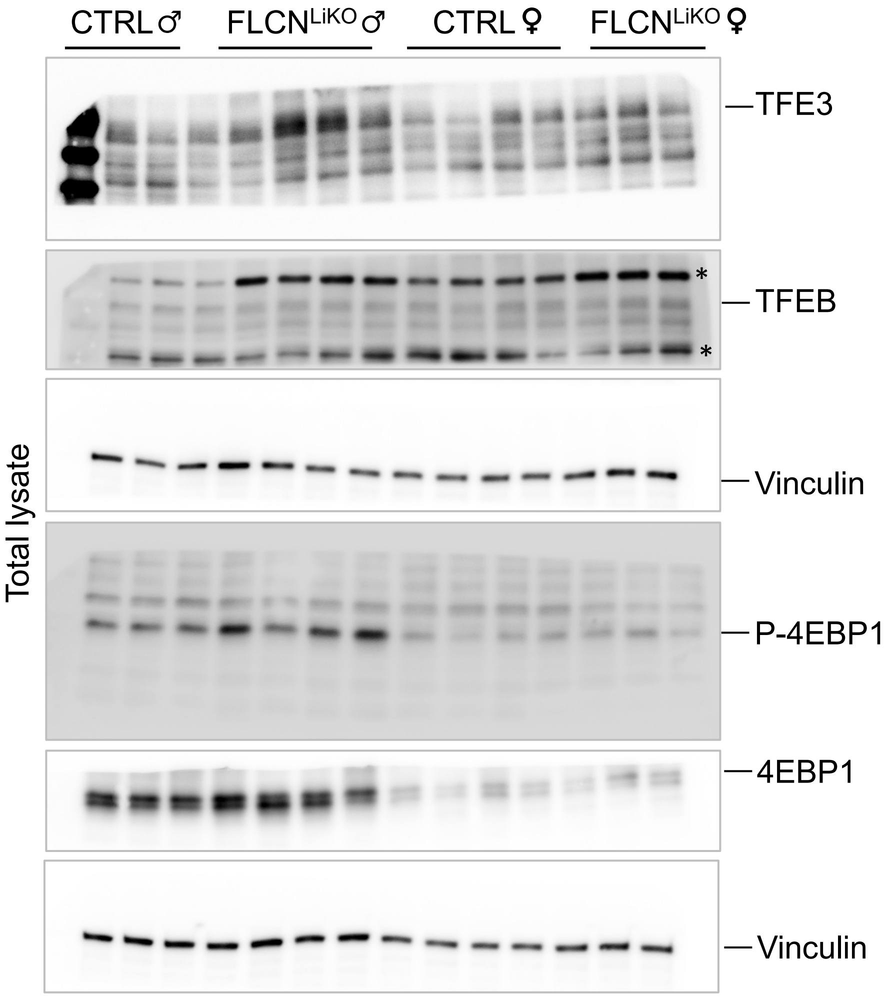


**B**

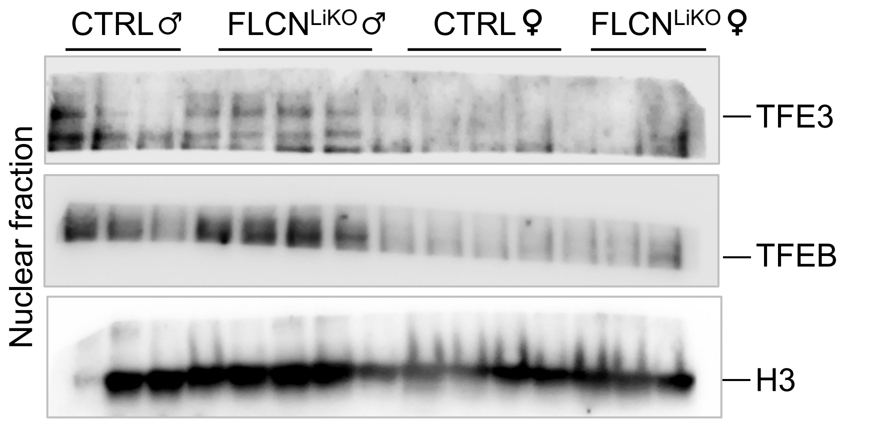

Supplement: Supplementary file 1 — Supplementary information [file 41418_2025_1486_MOESM1_ESM.pdf]
